# Layer-by-Layer Nanoparticle Assembly for Biomedicine: Mechanisms, Technologies, and Advancement via Acoustofluidics

**DOI:** 10.1021/acsanm.4c02463

**Published:** 2024-07-16

**Authors:** Seth Rowland, Amirreza Aghakhani, Richard D. Whalley, Ana Marina Ferreira, Nicholas Kotov, Piergiorgio Gentile

**Affiliations:** † School of Engineering, Newcastle University, Newcastle-upon-Tyne NE1 7RU, United Kingdom; ‡ Institute for Biomaterials and Biomolecular Systems, University of Stuttgart, 70569 Stuttgart, Germany; § Department of Chemical Engineering, University of Michigan, 2300 Hayward Street, Ann Arbor, Michigan 48109, United States

**Keywords:** Layer-by-Layer, nanoparticles, self-assembly, acoustofluidics, drug-delivery

## Abstract

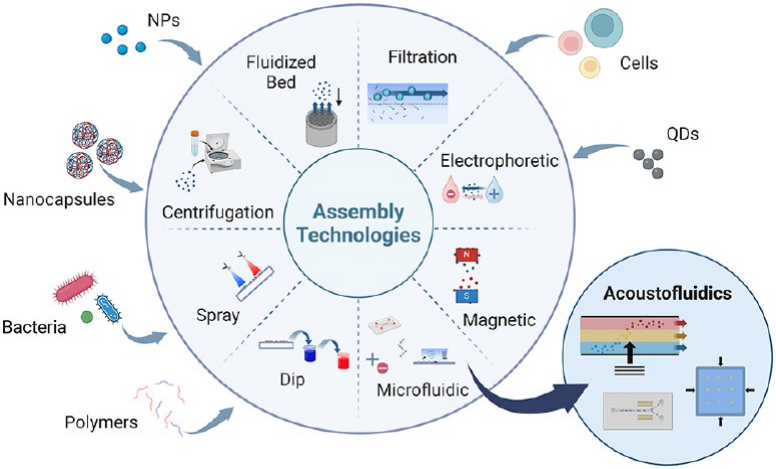

The deposition of thin films plays a crucial role in surface engineering, tailoring structural modifications, and functionalization across diverse applications. Layer-by-layer self-assembly, a prominent thin-film deposition method, has witnessed substantial growth since its mid-20th-century inception, driven by the discovery of eligible materials and innovative assembly technologies. Of these materials, micro- and nanoscopic substrates have received far less interest than their macroscopic counterparts; however, this is changing. The catalogue of eligible materials, including nanoparticles, quantum dots, polymers, proteins, cells and liposomes, along with some well-established layer-by-layer technologies, have combined to unlock impactful applications in biomedicine, as well as other areas like food fortification, and water remediation. To access these fields, several well-established technologies have been used, including tangential flow filtration, fluidized bed, atomization, electrophoretic assembly, and dielectrophoresis. Despite the invention of these technologies, the field of particle layer-by-layer still requires further technological development to achieve a high-yield, automatable, and industrially ready process, a requirement for the diverse, reactionary field of biomedicine and high-throughput pharmaceutical industry. This review provides a background on layer-by-layer, focusing on how its constituent building blocks and bonding mechanisms enable unmatched versatility. The discussion then extends to established and recent technologies employed for coating micro- and nanoscopic matter, evaluating their drawbacks and advantages, and highlighting promising areas in microfluidic approaches, where one distinctly auspicious technology emerges, acoustofluidics. The review also explores the potential and demonstrated application of acoustofluidics in layer-by-layer technology, as well as analyzing existing acoustofluidic technologies beyond LbL coating in areas such as cell trapping, cell sorting, and multidimensional particle manipulation. Finally, the review concludes with future perspectives on layer-by-layer nanoparticle coating and the potential impact of integrating acoustofluidic methods.

## 1 Introduction

Observing the surrounding environment reveals that every object or system exhibits a design that, originating from either human engineering or natural selection, has been tailored for specific environmental conditions. Notably, not all objects adhere to straightforward environmental prerequisites. In the context of biological systems, intricate behaviors are imperative to meet specific requirements, such as the successful delivery of drugs through human tissue for efficient absorption in biomedical applications.^1,2^ Addressing these challenges, Layer-by-Layer (LbL) assembly, an established technology, emerges as a promising solution.^3^ The LbL process, a self-assembly technique operating at the nanoscale, facilitates the creation of multilayer nanocoatings on a substrate to dictate unique behaviors.^4^ Essentially, this method involves the application of complementary solutions, wherein each layer accommodates the subsequent layer. Conventional demonstrations employ opposingly charged polyelectrolytic solutions, resulting in electrostatic interacting layers.

LbL proves to be a versatile technique capable of forming highly specific multilayer coatings by adjusting solutions and application parameters. Moreover, its applicability spans diverse scales, from hip implants^5^ to nanoparticles.^6^ This review focuses specifically on particle coating through LbL assembly, highlighting the method’s adaptability. The versatility of LbL becomes apparent when considering three pivotal factors, each offering numerous possibilities: materials (substrate and coating materials), assembly mechanisms (molecular bonding mechanisms in the self-assembly process), and the particle-LbL technique (the manner in which materials are exposed, influenced by advancements in LbL technology).

**Figure 1:**
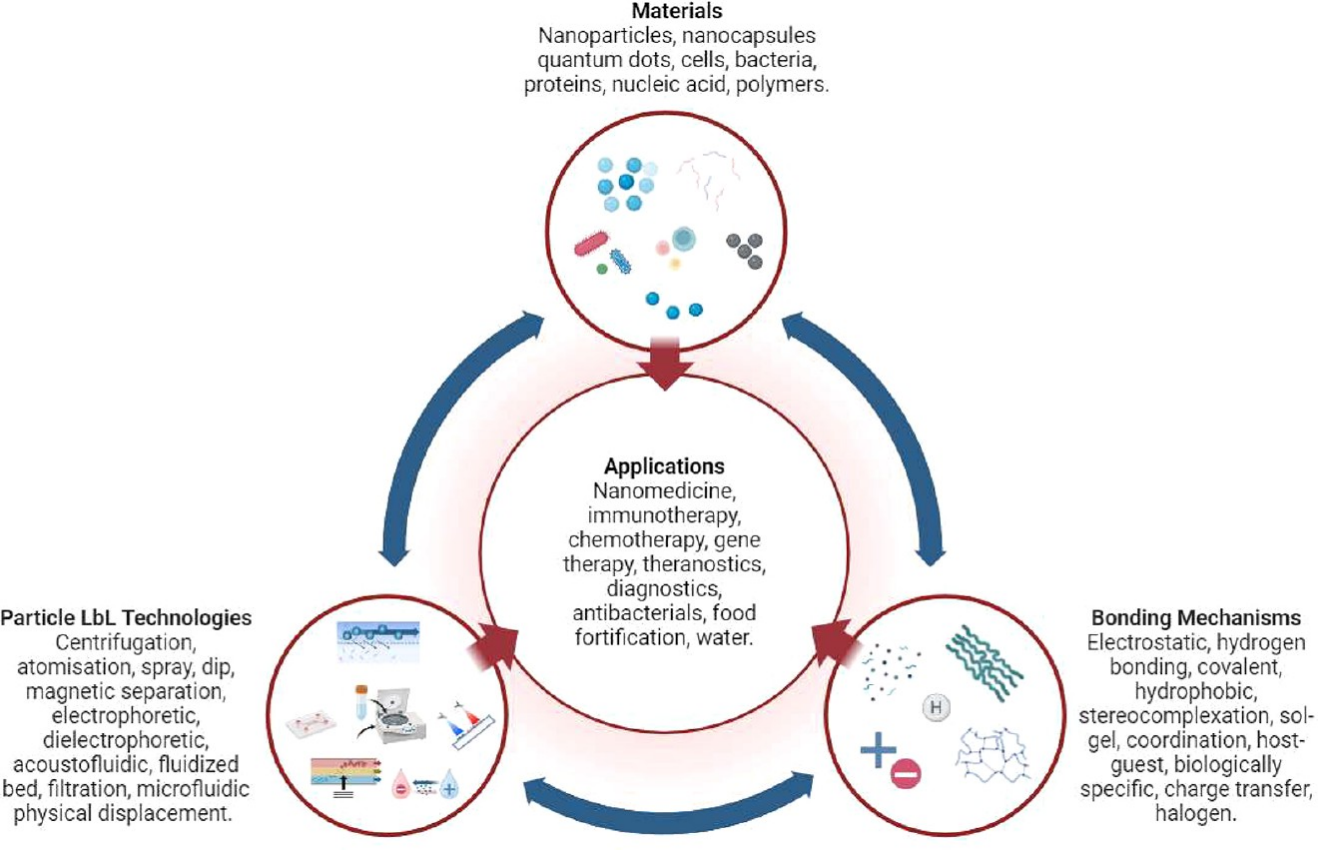
Relationship between materials, assembly mechanisms and particle-LbL technologies that enable novel applications.

These three factors exhibit mutual interdependence, wherein the introduction of new materials may necessitate or enable new assembly mechanisms, and vice versa. Similarly, innovative particle-LbL techniques may be prompted by changes in assembly mechanisms or materials. The continuous development in these three domains not only enhances the understanding of LbL but also opens avenues for novel applications of LbL-coated particles, particularly when considering new advances in nanoparticle formulation and drug delivery. This mutual interdependence is illustrated in Figure 1.

While numerous well-established techniques exist for coating macroscopic substrates, the literature on particle substrates remains comparatively limited, primarily due to the challenges associated with manipulation, particularly in the nano range. It is crucial to clarify that the term “nanoparticle coatings” in this context pertains to particles serving as the substrate for coating, rather than the adhesion of nanoparticles to a surface.

Despite the creation of numerous LbL particle formulations, their industrial adoption has been sluggish or nonexistent, primarily due to a lack of suitable technologies. Existing technologies, initially designed for lab-scale applications, fall short of meeting industrial requirements. The pharmaceutical industry stands out as a notable sector that could benefit significantly from LbL coating, given the challenges associated with the lengthy drug development timeline. Developing technologies for high-throughput, high-yield, continuous, and automated coating of nanoparticles could address these challenges and accelerate the drug development process. Two specific benefits in the pharmaceutical application include, (1) a wide range of nanodrug formulations can be taken rapidly from concept to reality, creating an opportunity to optimize said formulations at earlier stages of research, reducing time to clinical trials and expense; and (2) following trial and authoritative approval, the process can be used to deliver production quantities of novel drug formulations. Furthermore, the pharmaceutical market’s substantial size for nanoparticles in biotechnology, drug delivery and drug development, as evidenced by its reported value of $83.4 billion in 2020, with a projected Compound Annual Growth Rate (CAGR) of 8.2%, as reported by BCC Research LLC,^7^ engenders enthusiasm for LbL nanocoating technologies. Also, the applications of LbL particle coatings extend beyond pharmaceuticals to diagnostics, food preservation, and water remediation, amplifying the broader implications of developing new LbL technologies.

Multilayer coatings have come a long way from their inception; however, it is still useful to mention the original methods to highlight the issues that new technologies still sought to solve to this day. These inceptual procedures include the use of centrifugal purification steps, posing significant limitations: low throughput, aggregation of smaller particles, and an inability to be automated.^4,8,9^ Although appropriate for researching particle formulations and layering behavior at laboratory scale, these protocols can never be integrated into industry. As a result, numerous alternative technologies have been produced, such as atomization,^10^ fluidized bed,^11^ and tangential flow filtration,^12,13^ among others. By examining these developments, their drawbacks and their advantages, the most promising methodology, that is the focal point of this review, is illuminated: acoustofluidics. Acoustofluidics, in the broadest sense, is a combination of acoustics and microfluidics. Acoustics is conventionally the means to manipulate particles (and sometimes fluids depending on the application of the technology), while microfluidics provides the medium and environment for the acoustics to act. However, the dual approach of integrating microfluidics and acoustics is not the only form of microfluidics to appear in particle coating. Indeed, microfluidics enables high control, continuous throughput, waste and reactant minimization, and its incorporation has been demonstrated in other particle coating procedures, like in combination with electric fields in dielectrophoresis,^14^ magnetic fields in micromagnetofluidics,^15^ and hydrodynamic/inertial methods.^16^ These other techniques, that will be described in this review, present their own specific advantages and disadvantages, but without the versatility and potential application promised by acoustofluidics. It is also worth noting that recent reviews in the field of LbL reached similar perspectives that are echoed here, specifically the need for automated preparation methods of LbL particles to attain critical reproducibility and process streamlining.^17^

This literature review advocates for an in-depth exploration of LbL microfluidic particle coating. It will commence with a foundational understanding of the LbL process, encompassing bonding mechanisms and early centrifugal protocols. Subsequently, the review will delve into existing particle coating methods and evolving technologies, culminating in a detailed examination of acoustofluidics in LbL coating, comparing it with alternative methods. The versatility of acoustofluidics will be underscored by exploring its applications beyond LbL coating in various particle manipulation contexts. Finally, the review will assess the applications of LbL particles, focusing on bioengineering and drug delivery, offering critical insights into leveraging acoustofluidics for advancing or refining LbL particle technology.

## 2 A Background to Layer-by-Layer

### 2.1 Overview

LbL is a prominent method for functionalization of particle matter and continues to be well reviewed by researchers in the biomedical fields, a respectable feat for a methodology that was initially conceived over 50 years ago. The inception of the LbL could be argued to date to the 1960s, in which multilayers were formed on colloidal matter;^18^ however, the term LbL was not coined until the rise in popularity of self-assembly of multilayer structures in the 1990s.^19,20^ LbL has retained its popularity due to the inherent simplicity, low-cost, and versatility that is being continuously propagated by the discovery of new fabrication technologies, ensuring a continued enthusiasm for its use as nanofabrication technique.

**Figure 2:**
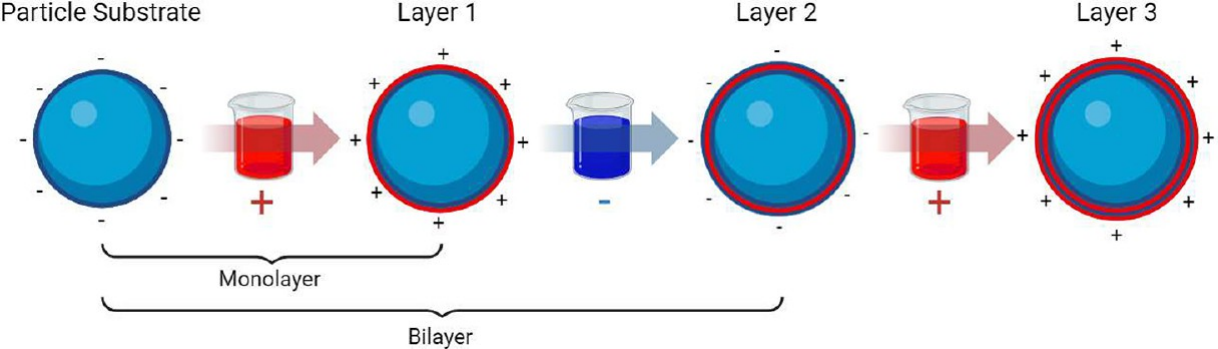
An arbitrary LbL process, starting with a negatively precharged particle substrate.

The LbL process necessitates alternating exposures of coating materials to a substrate. Following each material adsorption, the complementary function of the current layer and the desired following layer establishes the foundation for self-assembly. This iterative process is repeated to achieve a specified number of monolayers or bilayers, enabling tailored layer thickness and composition. Notably, a diverse range of LbL building blocks has been incorporated, encompassing polymers, peptides, carbon nanotubes, clays, dyes, metal oxides, enzymes, viruses, and nucleic acids.^21^ Furthermore, applications may necessitate the incorporation of more than two types of layering materials, allowing for the construction of multilayer systems with three, four, five, or any desired number of materials. This is contingent upon the repetition of the complementary bonding mechanism pattern, often anionic-cationic in electrostatic LbL, in accordance with the chosen bonding mechanism. Figure 2 illustrates the application of three monolayers, where cationic and anionic polymer solutions are used and the particle possesses an initial negative precharge. While electrostatic interactions remain central in LbL, alternative interlayer bonding mechanisms, including covalent,^22^ hydrogen bonding,^23^ and host–guest interactions,^24^ have been proposed in the literature and will be detailed in the subsequent section. Furthermore, recent developments in using the LbL self-assembly method to study electrode kinetics through NP and multiply electrode systems have been facilitated by novel in situ ligand exchange LbL.^25−27^

### 2.2 Bonding Mechanisms

Although the focus of this review is of particles specifically, the bonding mechanisms will be explained regardless of the substrate type. This is to say that while some mechanisms are not seen to be applied to particles in the literature, the theory would support their adaptation into particle coating and hence their inclusion in this review. Table 1 condenses the bonding mechanisms used in LbL self-assembly.

**Table 1:** Bonding Mechanisms For Building LbL Structures

*Mechanism*	*Driving the assembly*	*Eligible materials*	*Factors in layering behavior*
*Electrostatic*	Electrostatic attraction.	Anionic and cationic solutions with an initially charged substrate.	pH,^28^ temperature,^30^ ionic strength and electrolyte species,^29^ solvent quality,^96^ charge density and architecture,^34,97^ and molecular weight.^98^
*Hydrogen Bonding*	Dipole–dipole attraction.	Hydrogen acceptors and donors, often using the hydroxyl group (with alternatives using carboxyl, styrene, PAA, PVPh).^39^	Hydrogen donating and accepting groups.^99^
*Covalent*	Covalent bonding.	Covalently reactive polymer solutions and/or in addition to cross-linkers.	Temperature, salt-, reagent-, and cross-linker concentration, pH, reaction period.^52^
*Stereocomplexation*	van der Waals forces.	Highly structured synthetic stereoregular polymers carrying sites of opposing chirality.^100^ This has been demonstrated in addition to other bonding mechanisms, like electrostatic.^101^	Reagent concentration, molecular weight.^102^
*Coordination Driven*	Metal–ligand coordination bonding (coordinate covalent bonding).	Highly specific molecular combinations that behold the metal–ligand coordination groups^103^ that undergo acid–base reaction to form metal organic frameworks (MOFs).	Nature of the solvent,^104^ pH,^105^ temperature,^106^ the coordination environment of metal ions,^107^ steric constraints.^108^
*Host–Guest*	Highly specific host-bearing and guest-modified polymer bonding.	Host molecules include cucurbiturils, calixarenes, crown ethers and porphyrins and guest molecules including ferrocene, adamantane, and azobenzene.^109^	pH and ionic strength.^110^
*Biologically Specific*	Driving assembly is specific biological species dependent.	Biological matter, for example antibody–antigen,^111^ lectin–carbohydrate,^112^ and DNA hybridization.^39,113^	pH^114^ and ionic strength.^115^
*Charge Transfer*	Electron donor–acceptor exchange of groups on polymer side chains.	Nonionic polymers in which the donor and acceptor groups can be found at the ends of the polymer side chains, with strong accepting and donating groups.^76^	Temperature, concentration, and growth time.^116^
*Hydrophobic Interactions*	Entropy effect of nonpolar solutes destroying hydrogen bonds between water molecules.^117^	Hydrocarbons,^118^ fatty acids,^119^ aromatic compounds,^120^ nonpolar amino acids,^121^ and protein regions.^122^	Hydrophobic group density,^123^ temperature,^118^ pH,^124^ solvent properties.^125^
*Halogen*	Attraction of electron-deficient, covalently bonded halogens and nucleophilic sites in an electron donor–acceptor exchange.^126^	Halogens (iodine, bromine, chlorine), dihalogens, interhalogens, organic halides, halocarbons.^92,127^	Temperature,^127^ solvent properties,^128^ pH.^129^
*Sol–Gel*	Hydrolysis and condensation processes enabling inorganic polymerization.	Precursors: metal alkoxides (metalorganic compounds) with an organic ligand attached to a metal or metalloid atom.^130^	Temperature,^131^ pH,^132^ solvent properties,^133^ gelation catalysts.^134^

#### 2.2.1 Electrostatic Bonding

The most popular mechanism for LbL self-assembly uses electrostatic interactions, where an initially charged substrate is coated in complementary media. By altering the concentration of the coating media,^20^ pH,^28^ ionic strength,^29^ and temperature,^30^ as well as external stimuli such as an electrical field,^31^ light,^32^ and mechanical stress,^33^ electrostatic LbL can control the thicknesses, porosities, degradability, and reactivity of the multilayered structure. While it could be easily theorized that the alternation of complementary polyelectrolytes could go on indefinitely, in reality this appears to not be the case, where, for successful adhesion, a minimum molecular charge quantity is required.^34^ This becomes more difficult to satisfy as the number of layers in increased and the net surface charge moves to zero.

**Figure 3:**
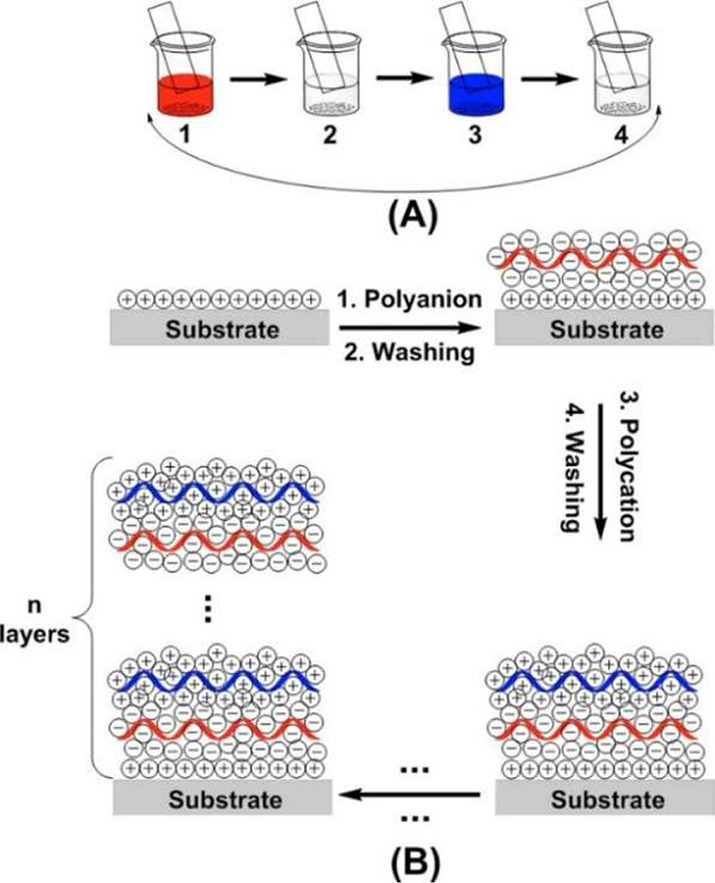
A) A schematic of immersion LbL of a large nonparticle substrate and B) A flow process showing the buildup of electrostatically charged layers. Reproduced with permission from ref (39). Copyright 2014, American Chemical Society.

The formation of layers can also follow different growth rates; exponential or linear. These layering relationships are largely determined by the coating media, the ionic strength of the solutions, and pH.^35,36^ In accordance with the development of the LbL principle, there has been a focus on the growth behavior of polyelectrolyte multilayers (PEMs), which extended to the linear and exponential growth regimes. For example, strong polyelectrolyte pairs like poly(styrenesulfonate) (PSS) and poly(allylamine hydrochloride) (PAH) show a linear growth relationship.^37^ In contrast, coating material combinations that contain polypeptides or polysaccharides demonstrate exponential layer growth.^38^ An arbitrary electrostatic LbL process is described in Figure 3.^39^ Recent studies in electrostatic LbL have included Pickering emulsion stabilization,^40^ small interfering RNA (siRNA) delivery mechanisms,^41,42^ water dye removal,^43^ and continued studies into drug release and stability,^44,45^ among others.

#### 2.2.2 Hydrogen Bonding

**Figure 4:**
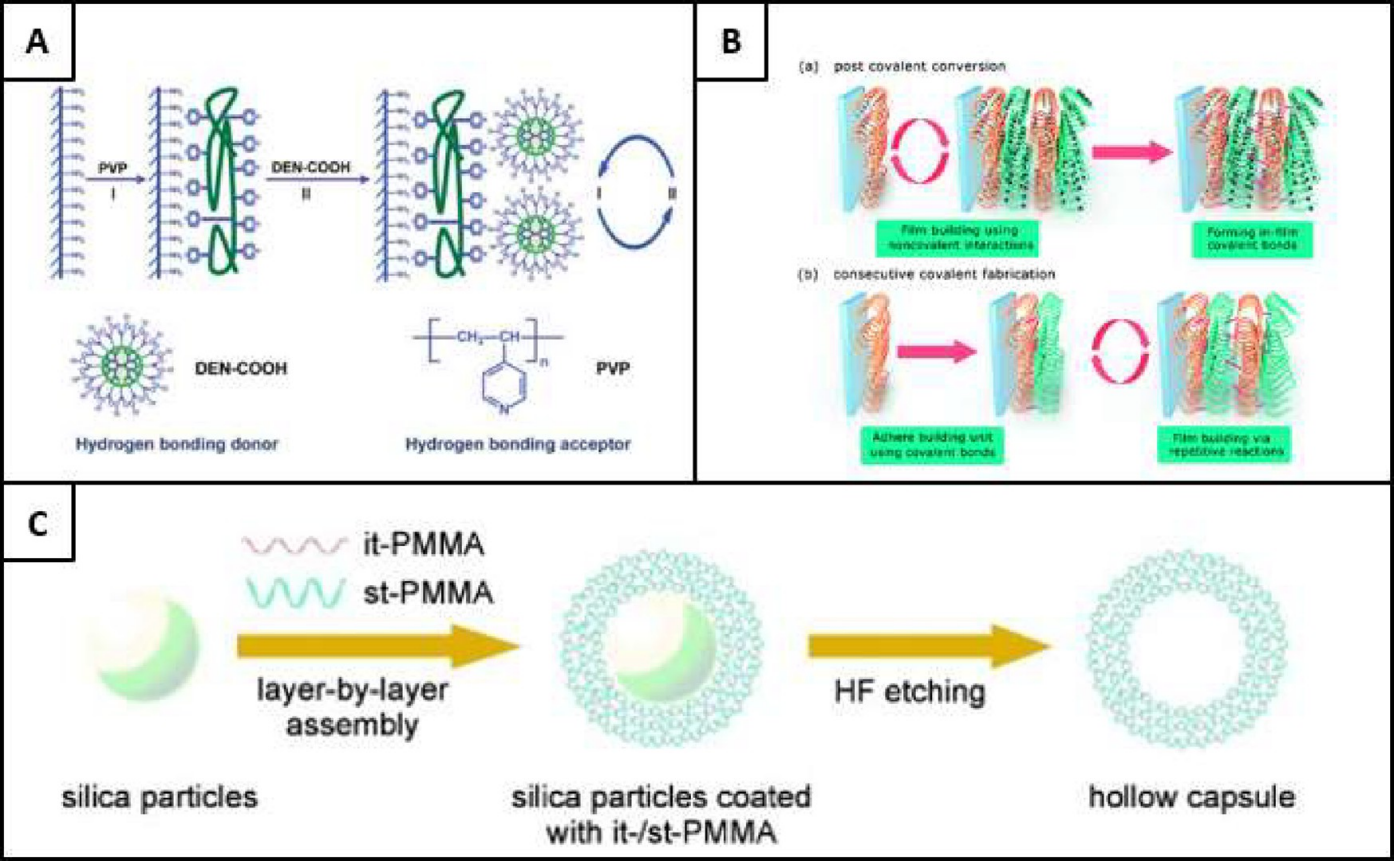
A) Schematic illustration of a hydrogen bonding LbL process, using a hydrogen bonding donor of a polyether dendrimer (DEN-COOH) and a hydrogen bonding acceptor of poly(4-vinylpyridine) (PVP). Reproduced from ref (46). Copyright 2003 American Chemical Society. B) A buildup of layers formed via covalent cross-linking, showing a) post covalent conversion and b) consecutive covalent fabrication. Notice the cross-links shown in pink, between adjacent polymer layers. Reproduced with permission from ref (52). Copyright 2018 Royal Society of Chemistry. C) Illustration of the fabrication process of it-/st-PMMA stereocomplex hollow capsules. Reproduced with permission from ref (63). Copyright 2006 WILEY-VCH Verlag GmbH & Co. KGaA Weinheim.

A second molecular mechanism for the formation of multilayer coatings is hydrogen bonding, in which complementary molecular groups (donor and acceptor) are presented, as illustrated by Figure 4A.^46^ This methodology was initially conceived in the mid to late 1990s, using a combination of polyaniline (PANI) and nonionic water-soluble polymers.^47^ Similarly, to electrostatic bonding, there are parameters involved in the coating process that influence the layer properties, like pH and polymer molecular weight.^47^ The fragility of the hydrogen bond to external stimuli like pH, ionic strength, and electrical fields has been studied and utilized in the development of erasable polymeric multilayers.^39,48,49^ However, when the bond is desired to be stronger, studies have also shown the use of cross-linkers working in addition to hydrogen bonding,^50^ demonstrating the versatility of hydrogen bonding as a mechanism. Hydrogen bonding continues to be a prevalent mechanism for LbL particles, with recent demonstrations of hydrogen bonding including controllable degradation of hollow mesoporous silica nanoparticles,^51^ as well as their adjacent use with electrostatic LbL in Pickering emulsions.^40^

#### 2.2.3 Covalent Bonding

Covalent bonding is another mechanism that continues to add versatility to the LbL landscape. Covalent cross-links between molecular chains can, via entropy driven chemical reaction, produce highly tailored structures.^52^ First discovered as a viable technique at a similar time to the discovery of hydrogen bonding LbL, covalently bonded LbL thin films were first created by using a complex of functional dendrimers and a reactive copolymer of maleic anhydride,^53^ with more recent studies in the early 21st century showing diphenylmethane derivatives with interlayer covalence occurring due to a urea component.^54^ Since this early development of covalent bonding LbL, other covalent complexes have been conceived like robust ultrathin films of oligoimide,^55^ fluorescent poly(ethylenimine) (PEI) nanotubes,^56^ hemoglobin protein hollow microcapsules^57^ and, in 2019, cationically modified membranes for antiviral drinking water applications.^58^ Covalent bonding as a mechanism is well review due to its simplicity, tunability, and applicability to a range of substrate types, such as planar, nonflat, micro, and nanoscale substrates.^52^ Where repetitive reactions using covalent bonds represents a conventional approach to covalent LbL, a second methodology also exists; post covalent conversion. Post covalent conversion refers to the process of forming covalent interactions from noncovalent interactions, where the noncovalent interactions include alterative bonding mechanisms, such as electrostatic interactions in electrostatic LbL. The difference between conventional consecutive covalent bonding and post covalent conversion can be seen in Figure 4B.^52^ Foreseeably, post covalent conversion is beneficial due to the elimination of interlayer reaction requirements, such as time and energy, like UV light irradiation conversion of ionic bonds^59^ and hydrogen bonds,^60^ but unfortunately places a prerequisite on the layering materials; the chains must possess a foundation bonding mechanism, which is not possible for all desired layering formulations. Whereas, consecutive covalent fabrication does not require alternative mechanisms of chain attraction and the intermittent layering can facilitate the incorporation of small molecules between chains.^52^

#### 2.2.4 Stereocomplexation Assembly

Stereocomplex assemblies involve highly ordered molecularly regulated polymeric structures. This method uses structurally ordered synthetic polymers that possess weak intermolecular van der Waals interactions, which form because of the prevalence of differential tacticities between polymers, rather than the tacticities between the same polymer chains.^4^ There has been little research since their discovery in the mid to late 1900s,^61^ where recent studies have primarily considered poly(methacrylates) and enantiomeric poly(lactides) deposition.^62^ In Figure 4C,^63^ a demonstration of isotactic (it) and syndiotactic (st) PMMA shows how a nonionic interaction between polymer chains can be used to form stereocomplex hollow capsules.

#### 2.2.5 Coordination Driven and Host–Guest

**Figure 5:**
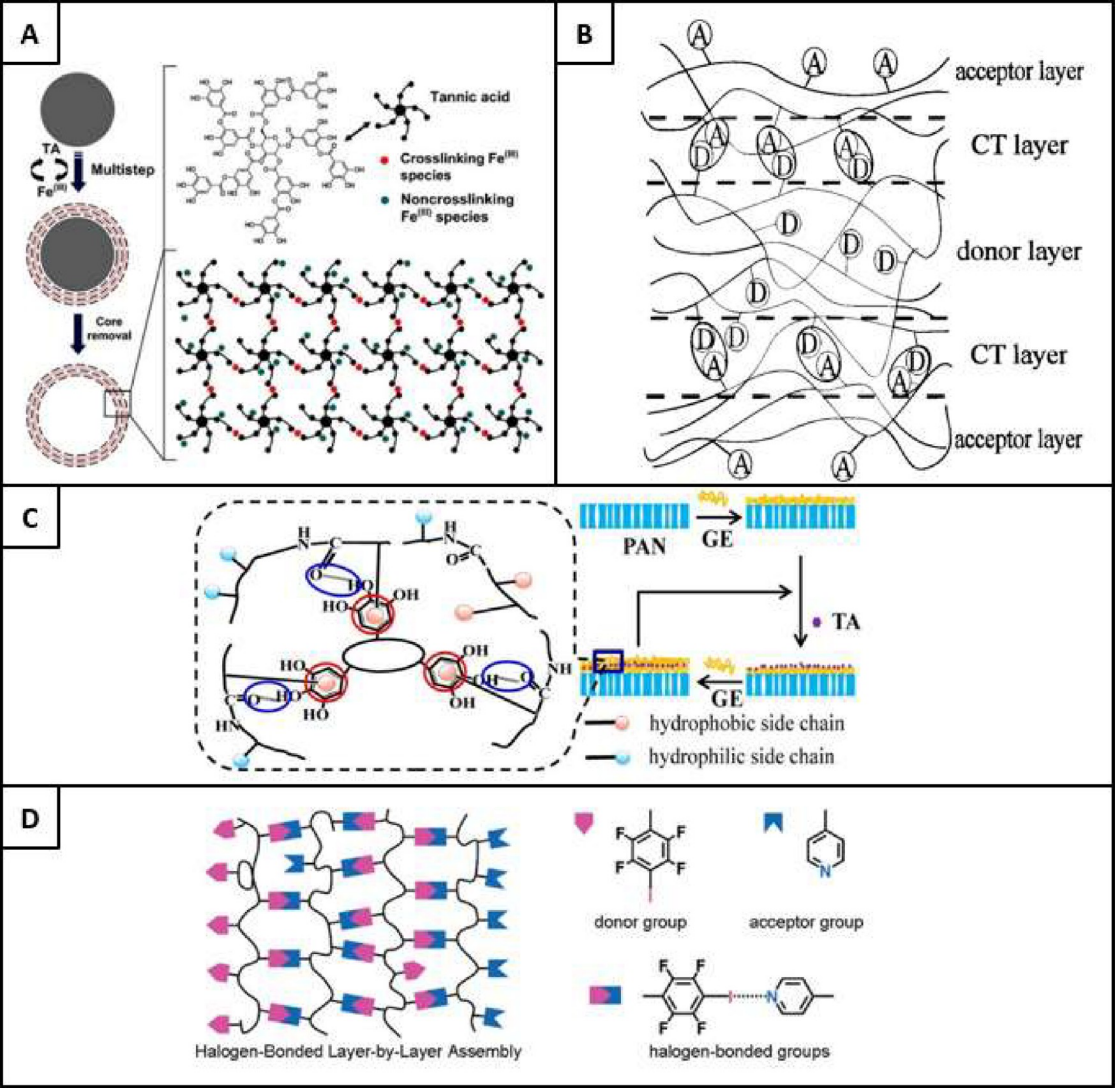
A) An illustration of the multistep layering assembly of tannic acid and cross-linking Fe^3+^ species, in the development of hollow capsules. Reproduced from ref (65). Copyright 2014 American Chemical Society. B) The interaction of acceptor and donor groups in the charge transfer bonding mechanism of multilayered structures. Reproduced from ref (76). Copyright 1997 American Chemical Society. C) The LbL process used in the formation of GE/TA ultrathin membranes, with an enlarged view of the molecular interlayer structure demonstrating a combination of hydrophobic interactions (red circles) supported by hydrogen bonding (blue circles). Reproduced from ref (81). Copyright 2013 American Chemical Society. D) The use of halogen bonding in a LbL assembly, with interactions formed due to the interaction of donor and acceptor groups. Reproduced from ref (95). Copyright 2007, American Chemical Society.

In contrast the weak van der Waals forces seen in stereocomplexation, coordination chemistry interactions are strong.^39,64^ These interactions occur between various metal ions and organic ligands, enabling the development of highly ordered versatile multilayer films and 3D architectures comprising of activated carbon, metal oxides, metal nitrides, zeolites, and advanced inorganic–organic polymeric films.^39^ An example of the assembly of a metal–phenol complex (MPC), using specific Fe^3+^ cross-linking species and tannic acid, is seen in Figure 5A^65^ in the production of multistep layered structures, which also have a subsequent core removal. With various applications in separation, sensing, membranes, drug delivery, luminescence, and super hydrophobic coatings, among others.^66−69^ This bonding mechanism has two routes for implementation, aqueous LbL and vaporous LbL. Another mechanism for LbL that can produce strong interlayer interactions is host–guest. For this method to work, similarly to coordination driven, highly specific molecule combinations must be used. These molecules include host molecules like cucurbiturils, calixarenes, crown ethers and porphyrins, and guest molecules like ferrocene, adamantane and azobenzene.^39^ Recent studies show host-guest interactions being used in the coating of living cells, specifically bone marrow mesenchymal stem cells (BMSCs), further demonstrating the versatility of LbL in its use of biological matter.^70^

#### 2.2.6 Biologically Specific

Biologically specific interactions depend on a high steric demand. These interactions are composites of other interactions that have been previously mentioned, like electrostatic,^71^ hydrophobic,^72^ and hydrogen bonding.^73^ However, the biologically specific nature is seen in the specific molecules that they target. Combinations of biologically specific multilayers include antibody–antigen,^74^ lectin–carbohydrate,^75^ and DNA hybridization.^39,73^

#### 2.2.7 Charge Transfer

Multilayer films can also be formed by using molecules that have an electron donor and acceptor relationship, known as charge transfer. The phrase charge transfer may induce the idea that the molecules are ionic; however, this is not the case. In reality, the terminology comes from a reaction taking place between complementary molecules, in which one species donates and the other accepts a charge. The region by which this takes place is termed the charge transfer (CT) layer, as seen in Figure 5B.^76^ This mechanism was first demonstrated in 1997.^76^ Since then, it has been used to bond multilayer complexes such as PAH and tetrasulfonated metallophthelocyanines (NiTsPc and FeTsPc) for electrochromic and sensing applications^77^ as well as thiol-functionalized tetrathiafulvalene derivatives (TTF-CH_2_SH) and 7,7,8,8-tetracyanoquinodimethane (TCNQ) on gold substrates.^78^ The complexes can also be prepared in organic solvents, increasing the incorporation of hydrophobic groups for making nanostructured films based on organic materials, with applications in electronics, photonics, and optics devices.^39^

#### 2.2.8 Hydrophobic Interactions

Hydrophobic bonds rely upon the hydrophobic nature of the layering materials, existing as a particularly important role for uncharged molecular combinations. A recent demonstration of hydrophobic LbL was seen in the deposition ofpoly(vinyl alcohol) (PVA) on a gold substrate, where the exposure of the substrate to a PVA aqueous solution and then drying with a stream of nitrogen gas was used to build a monolayer. By repeating this, multilayer complexes are formed.^79^ Kotov et al. also discovered the importance of hydrophobic interactions in polyelectrolytic LbL assembly, where the two phenomena combine in the layering procedure to deliver stability of interlayer adhesion.^80^ The interplay of two bonding mechanisms also includes that shown in Figure 5C,^81^ where hydrophobic interactions are reinforced by hydrogen bonds in the preparation of ultrathin gelatin (GE) and tannic acid (TA) membranes.

#### 2.2.9 Sol–Gel Mechanism

As the name suggests, sol–gel is a mechanism in which a solid (gel) material, specifically an inorganic integrated network, is synthesized from an initial number of small molecules in a liquid (sol), from solution to gelation. This sol–gel behavior was first demonstrated in 1845^82^ and has since undergone continued development to its incorporation into industry today.^83^ Simply, it follows a process of hydrolysis, disassociation of chemical bonds and condensation of precursors.^84^ These precursors mainly include metal and metalloid alkoxides, members of the metalorganic compound family, which have an organic ligand attached to a metal or metalloid atom.^85^ The use of sol–gel has been demonstrated in many macroscopic coating procedures, utilizing dip,^86^ spray,^87^ spin,^88^ and roll^89^ methodologies. As well as a documented proficiency in macroscopic applications, the sol–gel mechanism has also been shown to be useful for the coating of nanoparticles.^90^

#### 2.2.10 Halogen Bonding

Whilst halogen bonding was originally discovered in the 1860s, the specific characteristics and behaviors of halogen bonding were not studied for over a century later until 1970.^91^ Since then, then the incorporation of halogen bonding into LbL has been swift. Halogen bonds appear to mirror the behavior of hydrogen bonds, with both hydrogen atoms and halogen atoms behaving as electron acceptor groups. Contrary to the use of halogens as electron acceptors is the fact that they also act as hydrogen bonding groups, whereby all four halogens have been evidenced as donors, from the highest tendency to form strong interactions, iodine, to the weakest halogen bonding halogen, fluorine.^92^

With the evidencing of halogen bonding as a methodology for crystal engineering and supramolecular crystals,^93,94^ as well as the prevailing of halogen bonding over hydrogen bonding in a competitive recognition process, halogen bonding was soon realized in the field of coatings, as well as specifically in LbL, as is evidenced by Figure 5D.^95^ However, the use of halogen bonding in relation to the LbL procedure on particles is yet to be seen.

### 2.3 Importance of LbL Technology on Bonding Mechanism

As is demonstrated through the multitude of available bonding mechanisms, particle LbL self-assembly is a versatile technique. The bonding mechanism between these layers significantly impacts the selection and optimization of the technique employed for assembly, such as centrifugation, tangential flow filtration, fluidized bed, or microfluidic methods. The choice of bonding mechanism determines the stability and strength of the assembled layers, as well as the rate and efficiency of the assembly process. For instance, if layers are held together primarily by electrostatic forces, techniques that manipulate electrical properties, like electrophoresis or dielectrophoretic microfluidic methods, may be particularly effective.

Alternatively, when layers are bonded through hydrogen bonding, techniques that allow for gentle and controlled interactions, such as fluidized bed or microfluidic methods, may be preferred. These approaches provide a conducive environment for the formation and stabilization of hydrogen bonds without disrupting the assembled structure. Similarly, in cases where covalent bonds are formed between layers, techniques that facilitate chemical reactions, such as microfluidic methods or surface modification approaches, may be more suitable. These methods enable precise control over reaction conditions and promote the formation of strong and stable covalent bonds.

Within the microfluidic-sphere, acoustofluidics appears as particularly accommodating of the multitude of bonding mechanisms employed in LbL. As will be seen in Section 3, acoustofluidic methods utilize acoustic waves to manipulate particles or fluids, allowing for precise control over particle positioning and assembly. By adjusting parameters such as acoustic frequency, intensity, and fluid properties, this technique can be tailored to accommodate different bonding mechanisms. Additionally, acoustofluidic methods offer advantages such as scalability, compatibility with a wide range of materials, and the ability to operate under mild conditions, making them suitable for diverse applications and bonding mechanisms. Overall, the molecular bonding mechanism between layers in particle LbL assembly influences the choice and optimization of assembly techniques, with acoustofluidic methods providing a versatile and adaptable approach for facilitating precise and efficient LbL assembly processes.

### 2.4 Centrifugal Protocols

Before delving into an exploration of established and developing technologies in the realm of LbL, it is essential to elucidate the foundational procedure employed for particle coating. This method uses centrifugation as the process by which particles are separated from their medium following exposure to a new material.^8^

One of the most fundamental approaches to LbL coating on the macro scale is immersion, often referred to as dipping. Analogously, the centrifugal approach mirrors this macroscopic process, wherein particles (substrates) are essentially immersed in a coating solution—typically a polyelectrolytic solution for the electrostatic form of LbL. Precise control over material concentration, viscosity, temperature, and other properties allows for the manipulation of layer properties. After immersion in one charged material, particles need to be separated from the coating material solution through centrifugation. After successful separation, the particles undergo washing in a buffer solution, followed by centrifugation and the initiation of the subsequent layer by immersion into the next material (a polyelectrolyte of opposing charge to the previous one). This sequence is repeated to achieve the desired number of layers.

While this process is straightforward, utilizing basic equipment and easily executable in a laboratory environment, it encounters challenges when applied to coating particles below 10 μm and within the nanoparticle range. At this size, particle aggregation becomes a significant issue. With an increasing number of layers, the percentage of useful yield diminishes, rendering this approach expensive and wasteful for LbL coating. Another fundamental challenge arises in the scalability of this process to an industrial setting. Not only does the economic viability suffer due to aggregation losses at small particle sizes, but the automation of centrifugation proves to be a cumbersome task for industrial adoption.^8^

## 3 Layer-by-Layer Coating Technologies

Approaches for the application of the LbL process on particles have acquired research focus in the last couple of decades and the next section of this review is focused on the current LbL approaches proposed for the coating of particles with a critical perspective on the nanoscale size. Table 2 compares the available technologies for coating particle substrates.

**Table 2:** LbL Technologies Currently Used for Coating Particle Substrates

	*Technology*	*Overview*	*Advantages*	*Disadvantages*
*Spray*	Centrifugation	Sequential immersion and intermittent washing using centrifugation to separate particles and coating/washing media.	Requires basic equipment, well established methodology.	Low yield, manual, time intensive, unautomated.
	PRINT	PRINT manufactured particles exposed to sequential spray coating.	Industrial, repeatable, automated.	Particle type and size restricted by methodology, waste.
	Atomization	Solution atomization and coating media exposure through condensing path.	Simple, nanoparticle focused.	Low throughput and process control.
	Quasi LbL	Simultaneous spraying of coating media to produce polyelectrolyte complexes.	One-step simplicity.	May not be considered LbL, lack of control and reproducibility.
*Dip*	WETS	Dip coating templated particles, where wettable domains are the only coated area, minimizing waste.	Low waste, automatable and industrially eligible.	Particle type and size restricted by methodology.
*Microfluidic*	Dielectrophoresis	Dielectrophoretic forced migration of particles through coflowing laminar coating streams.	High-control, automatable.	Heating at high power, low control and throughput.
	Microfluidic Physical Displacement	Internal structures and bulk fluid motion combine to migrate particles through coflowing laminar coating streams.	Automatable, simple design and implementation.	Small particle incompatible, low control and throughput.
	Inertial Microfluidics	Changes in channel geometry expose particles to various coating media.	Automatable, simple design and implementation.	Poor media separation, low throughput.
	Acoustofluidics	Primary acoustic radiation force migrates particles in predetermined paths or specific locations exposing particles to coflowing laminar coating streams.	Automatable, label-free, biocompatible, versatile.	Low-throughput, restricted control due to fixed tilted angle architecture.
	Magnetic	Magnetic forces force particles through coflowing laminar coating streams.	Automatable.	Only suitable for magnetic particles.
*Filtration*	Membrane filtration	Particles are filtered intermittently from coating and washing media.	Simple design and implementation.	Membrane clogging, poor control.
	Tangential Flow Filtration	Particles are filtered intermittently from coating and washing media, but flow direction is tangential to membrane orientation.	Reduced clogging, reproducibility, well-controlled.	Low yield.
	Magnetic separation	Sequential immersion and intermittent washing using magnetic forces to separate particles from washing/coating media.	Simple setup and equipment, established methodology.	Not versatile, only suitable for magnetic particles.
	Electrophoresis	Particles in a porous substrate exposed to electrophoretic coating media flow.	Particle versatility, automatable.	Limited to charged materials only.
	Fluidized bed	Balanced gravitational and flow forces facilitate particle suspension and sequential exposure to coating media.	Low process time, high yield, scalable.	Not suitable for nanoparticles.

### 3.1 Spray Assembly

LbL spray assembly has been a cornerstone of LbL for large macroscopic substrates for decades, however more recent incorporations of spray assembly in the field of LbL have been focused in the microscopic and nanoscopic domain of particle matter. While the similarity in terminology may engender the impression that the processes are directly similar, the smaller substrate size requires an entirely different approach. The two established methods of spray LbL for particles include Particle Replication in Nonwetting Templates (PRINT), where particles are immobilized in their production vessels and subject to spraying, and aerosolization, in which they become airborne and navigate through environments that enable LbL coating. Furthermore, there are also methods that use spray principles in a field of LbL named Quasi-LbL. These different approaches will be presented in the following section.

#### 3.1.1 PRINT

**Figure 6:**
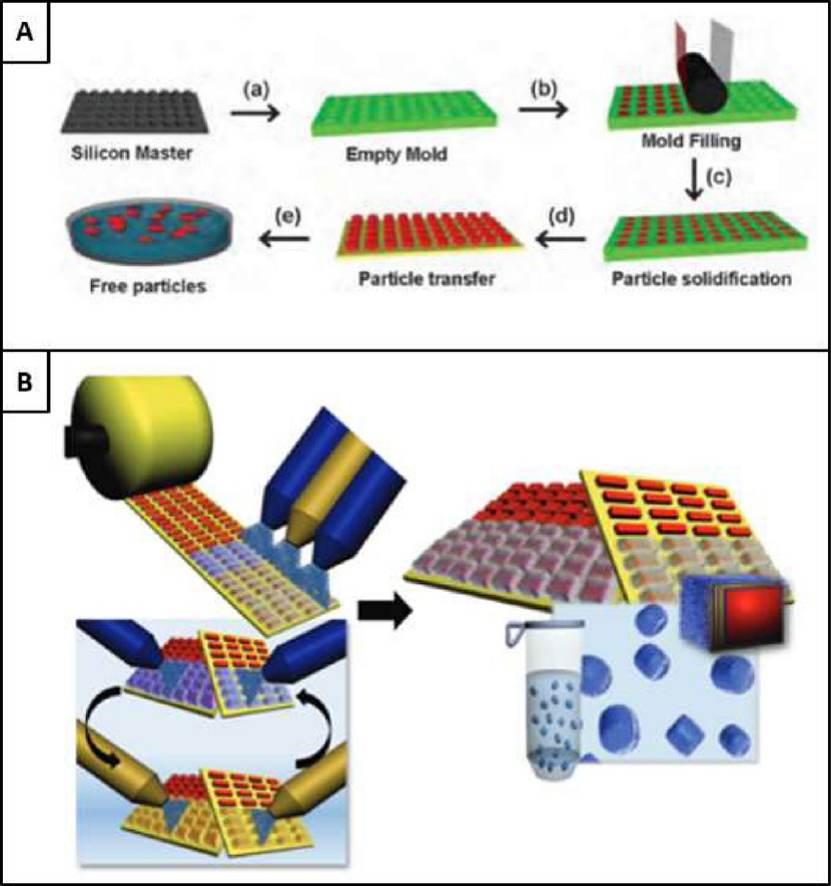
A) Print particle manufacture schematic. Reproduced with permission from ref (135). Copyright 2012 Royal Society of Chemistry. B) The PRINT particle LbL methodology of particle arrays on the harvesting layer undergoing spray LbL assembly. Reproduced with permission from ref (136). Copyright 2012, WILEY-VCH Verlag GmbH & Co. KGaA Weinheim.

PRINT refers to particles being produced from a specific manufacturing methodology, which produces particles in the range of 50 nm to 200 μm in a monodispersed format with specific geometries. The technique uses molds which are filled with the relevant particle matter before being rolled flat on the surface of the mold, solidified and transferred to the harvesting layer.^135^Figure 6A shows the manufacturing schematic for PRINT particles.^135^ While this process itself is not LbL, LbL spray techniques are used consequently to the particle manufacture. With roll-to-roll modularity, particles are exposed to the complementary solutions necessary to produce to LbL multilayer coatings as seen in Figure 6B.^136^

This methodology is particularly advantageous in that it meets the demands for throughput required by industry, attaching the LbL aspect to the PRINT particle manufacturing process, a particle manufacturing process that delivers incredibly well-defined, precise particles in a highly scalable approach. However, this process does not account for the versatility that many particle coating research applications may require, versatility that is inherent to LbL, like personalized precision drug-delivery.^137^ Furthermore, this methodology requires considerable costly overheads since it is linked, chained, and hence restricted by the antecedent PRINT particle manufacture, requiring a bespoke mold, integrated to the production process, for every formulation or particle type desired and cannot produce geometries without a rolled-flat edge. Lastly, this process is also wasteful due to the empty geometry between particles also being coated with expensive drug-containing materials.^138^

#### 3.1.2 Atomization

**Figure 7:**
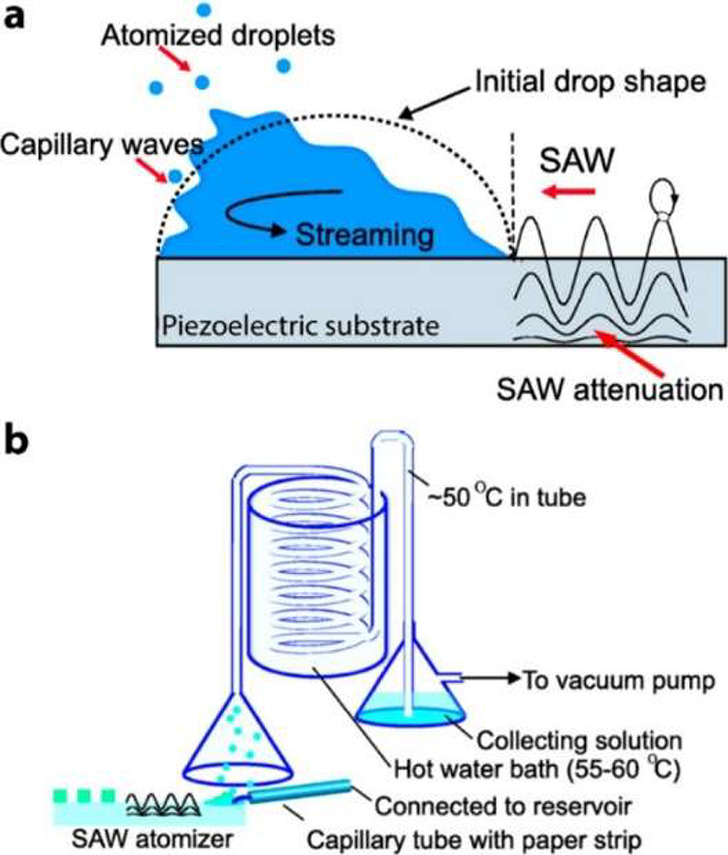
Production of atomized nanoparticles, a) the atomization of an initial droplet due to SAWs on a piezoelectric substrate and b) the apparatus and procession of the atomized droplets, evaporation of solvents and then final collection of nanoparticles. Reproduced from ref (10). Copyright 2011 American Chemical Society.

While other LbL methods require an original template, there have been interesting developments in template-free synthesis of LbL particles. Specifically, the atomization technique uses surface acoustic waves (SAWs) to atomize an aqueous solution of polymeric excipients or proteins. Once atomized, the airborne particles enter a process designed to evaporate the carrier solvents. The remaining particles are collected in a bath.^10^ This process is considered a spray process because the active particles are part of the spraying material, rather than being held stationary while the coating media is sprayed unto them, as seen in previous spray methodologies. This initiates the process by which particles can be LbL coated. Then, the LbL process is implemented by the polyelectrolytic solutions that the particles are collected in and acts as the aqueous solutions for the consecutive atomization. Figure 7 shows the scheme of the atomization process, that can be used to create and coat particles in the order of 10–100 nm.^10^ However, this process is restricted by its usefulness for larger particles, low throughput for industrial applications due to small SAW atomizing devices, lack of control throughout on the coating process and hence variability in output layering characteristics.

#### 3.1.3 Quasi-LbL Approach

**Figure 8:**
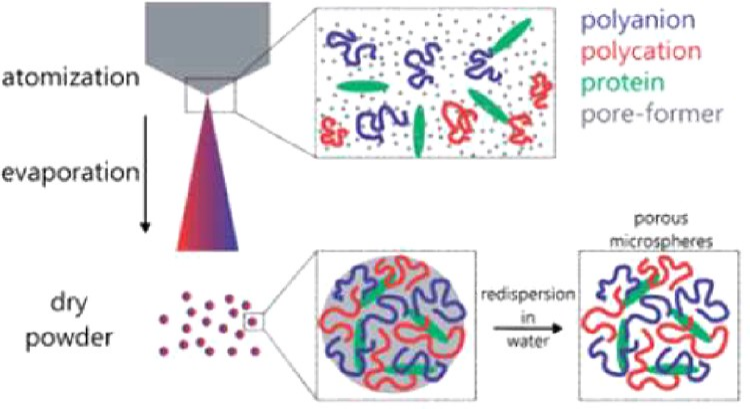
A schematic of the generation of quasi-LbL coated particles. Reproduced with permission from ref (141). Copyright 2014 Royal Society of Chemistry.

A related, but not strictly LbL methodology for spray assembly is the quasi-LbL, that has been demonstrated on macroscopic large planar substrates,^139^ but also been shown to be useful for particle substrates.^140^ In the previous paragraph, an atomizing process was described that used SAWs to layer particles, quasi-LbL of particles follows a similar protocol. This protocol is shown in Figure 8.^141^

The coating solution, a pore former (called coprecipitation agent) with incorporated biomolecules, are all simultaneously atomized, often from a spray nozzle, and then evaporated to leave polyelectrolyte complexes.^140^ This methodology is considered a recent development and hence has limited research and even more limited applicability to LbL considering its necessity for coincidence of solutions, where LbL would more strictly concern itself with distinct, separate exposure cycles. However, the ability to produce interesting new films should be regarded as an important capability of this method, that would be fortuitous to find in other LbL coating technologies.

### 3.2 WETS

One of the more recent advances in LbL particle fabrication is in a dip methodology, sometimes referred to as immersion assembly. Similarly to that which is described for spray assembly, dip assembly usually concerns itself with macroscopic methodologies, being a well-established technique for coating large planar and nonplanar substrates. However, dip coating had, until 2019, only retained use in microscopic particle coating as a step involved in the centrifugation methodology. This changed upon the invention of wettability engendered templated self-assembly (WETS).

**Figure 9:**
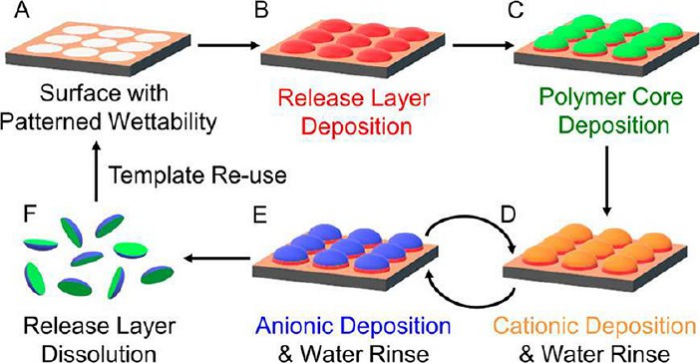
Schematic of the WETS process that incorporates polyelectrolyte (LbL) deposition on top of neutral polymeric particles, where A) patterning of wettable surface, B) deposition of release layer, C) deposition of polymer core, D) cationic deposition, E) anionic deposition, where depositions include a water rinse and can be repeated to a desired regime, F) dissolution of release layer. Reproduced from ref (138). Copyright 2019 American Chemical Society.

Referring to spray methodologies earlier, WETS is like PRINT particle LbL but tries to improve upon the losses associated with spray assembly and lost material in the empty geometries of PRINT particle LbL. The process uses wetting domains on a TiO_2_ surface to determine the particle shape, then using a sacrificial release layer and a polymer core deposition the foundation for the LbL process is set. Detailed only for polyelectrolytic deposition, the LbL process involves the immersion of the particles in alternating solutions. Following the desired layering regime, the particles are released from the TiO_2_ surface by dissolution of the release layer.^138^Figure 9 depicts the WETS process.^138^

While the WETS technique solves many of the issues associated with PRINT particle LbL, cleverly reducing waste by ensuring that coating materials only accumulate over the wettable domains, it does not solve the issues with LbL particle coating technologies comprehensively. However, it would appear that WETS was not envisioned as replacement for LbL techniques, but rather a methodology for producing Janus particles, to enable dual release of multiphasic polymeric drug formulations. As such, WETS sits on the fringes of what can be considered LbL, serving well as a demonstration of how immersion can be used to eliminate waste but not attaining the applicability required for a LbL process due to the intrinsic requirement for preceding particle substrate manufacture in the same process, reducing the versatility of the presented LbL technology.

### 3.3 Magnetic Separation

To mention magnetic separation as a methodology for LbL coating may be considered unnecessary. However, this method does help solve problems found in the primordial method of centrifugation. While magnetic separation itself does not present a new methodology to coat particles, it does exist as a useful alternative to segregation and the problems, like aggregation and low throughput, that exist with centrifugation between exposure of the particles to coating materials. Magnetic separation involves either loading the emulsions^142^ or the particles^143^ with magnetic nanoparticles. Despite the advantages of this technique to avoid issues surrounding centrifugation, the method does not itself present a useful way to coat particles and it relies on the addition of foreign magnetic bodies. While a useful method, magnetic separation is not considered to solve the problems necessary to produce a continuous, versatile particle coater.

### 3.4 Electrophoresis

**Figure 10:**
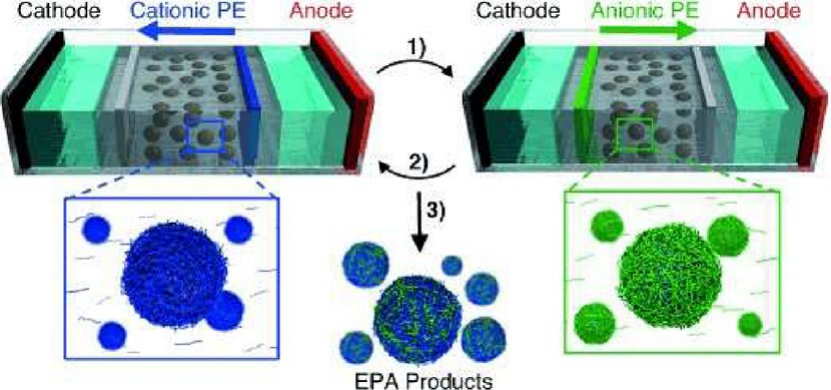
Schematic illustrating the use of EPA. Process 1) A cationic solution is loaded into the walls adjacent to the anode, moving through the agarose and consequently coating particles, process 2) showing the loading of an anionic polyelectrolytic solution into the walls adjacent to the cathode and passing through the agarose under the electrophoretic force, coating the particles once more, and process 3) heating of agarose to recover the EPA products. Reproduced with permission from ref (145). Copyright 2013 WILEY-VCH Verlag GmbH & Co. KGaA Weinheim.

Electrophoresis (EP) refers to the movement of charged matter due to a uniform electric field.^144^ The physics were originally used in LbL in the form of an electrophoretic assembly (EPA) method.^145^ It is worth noting here that the general rules for electrophoresis are not sufficient in describing the implementation of the method. In the following example the particles, although charged, do not move, and instead it is the solution that is migrated under the electrophoretic force. The particles are suspended in a porous substrate, usually agarose. LbL coating is generated by the lacing of walls adjacent to the electrodes with cationic and anionic polyelectrolytic coating solutions. When the electrodes are turned on, this causes the migration of either the anionic or cationic polyelectrolyte solutions through the porous agarose and suspended particles, thus coating them. Figure 10(145) describes this methodology.

This method is useful in addressing many of the issues seen in the centrifugation methodology and has been demonstrated on a vast range of particle sizes, from 35 nm to 3 μm. This method also shows promise in the possibility for automation; however, this is not still demonstrated in reported literature.^145^ One of the major drawbacks over this method is that it relies upon charged particles and solutions, a distinct disadvantage when considering the various, uncharged bonding mechanisms available to the LbL, described in the preceding sections.

### 3.5 Fluidized Bed

**Figure 11:**
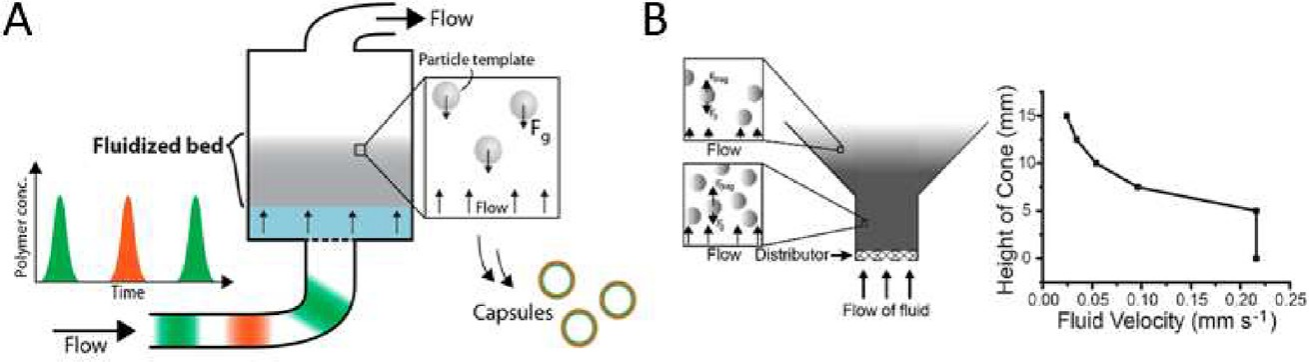
A) The fluidized bed method for LbL deposition to produce capsules, where F_G_ represents the gravitational force on the particles (Reproduced from ref (146). Copyright 2014, American Chemical Society), and B) the improved method for smaller particles sizes, where F_drag_ is the drag force exerted on the particle by the fluid flow and F_g_ is the gravitational force (Reproduced from ref (147). Copyright 2015, American Chemical Society).

The first demonstration of a fluidized bed method being used to produce multilayered capsules was published in 2014.^146^ The fluidized bed suspends particles in water by regulating the flow rate. This flow rate determines the drag force on the particles and balances this with the gravitational force. By suspending them in this format the coating solutions and media can be added via the flow, passing over the suspended particles, hence producing the desired multilayer capsules, as described in Figure 11A.^146^ A major aim of the development of this technology was to decrease the time taken in the previous technologies to coat particles and to increase the viable yield of the available processes. The methodology successfully achieved this with a 30 min per batch production rate, 98% viable particle yield, scalable process, demonstrated on 50 μm particles by Richardson et al.^146^ Unfortunately, although the process can theoretically be used for smaller particles, the drag force on the particles exerted by the flow would require considerable reduction, necessitating extensively low flow rates.^146^ The decrease in flow rate means that the time taken to coat the particles becomes like that of the previous methods, making it unviable for smaller particles if industrial and clinical applications are intended. This issue was somewhat rectified by the development, by the same research group, of a tapered fluidized bed profile. This meant that as the cross-sectional area increased (as shown in Figure 11B),^147^ the velocity of the bed would decrease, decreasing drag and hence enabling small particles (∼3 μm) to be coated.^147^ Unfortunately, this still fails to cater to particles in the nano range, which have more applications in popular research areas like in biomedicine and drug delivery.^148^

### 3.6 Microfluidic Techniques

Microfluidic methods for LbL coating have had considerable interest in recent years, due largely to their intrinsic high degree of control during the material-particle exposure and exhibiting continuous processing characteristics.^149^ However, there are some drawbacks to the conventional microfluidic approaches including low throughput,^150^ which make them less adoptable into industrial and clinical applications. The following section sorts to define the existing methods and how each have been designed to overcome issues of conventional LbL processes. Whilst the following approaches include passive methdologies like flow displacement and pillaring, this section also includes active methods (integrating external stimuli), like dielectrophoretics, acoustics, and magnetics. The inclusion of both active and passive methodologies under the umbrella of microfluidics is enabled by thier reliance on the same fundamental microfluidic phenomena, laminar flow regimes.

#### 3.6.1 Microfluidic Dielectrophoresis

Dielectrophoresis (DEP) is often confused with EP due to the similarities in their practical and physical requirements. Both require the use of electrodes, and both manipulate particles with electric fields. However, the main difference is the mechanism in which particles are deflected by the electric fields: uniform fields in EP while nonuniform for DEP. The nonuniform electric field in DEP acts upon the difference in polarizability of the particle in respect to its solution. The two types of DEP are positive-DEP (p-DEP), where the particles move toward the region of high electric field gradient, and negative-DEP (n-DEP), where the particles move toward the lower electric field gradient.^151^

**Figure 12:**
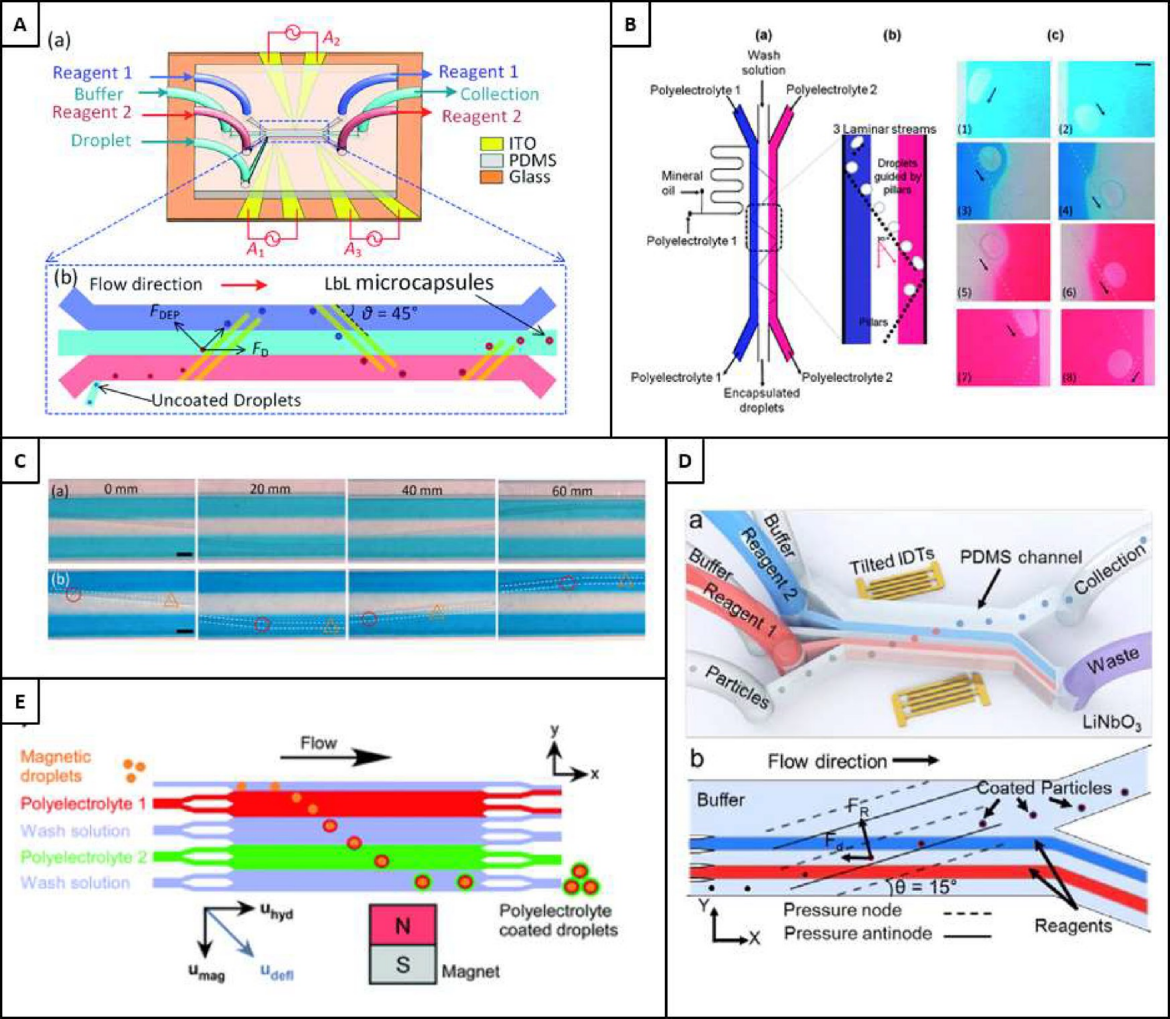
A) DEP Microfluidics where a) a schematic illustration of the setup of the DEP particle coating device and b) a blown-up view of the microchannel and electrode setup, as well as the procession of particles as they pass down the channel where F_DEP_ is the dielectrophoretic force and F_D_ is the drag force exerted on the particles by the fluid. Reproduced with permission from ref (14). Copyright 2021 Royal Society of Chemistry. B) The microfluidic pinball technique, where a) entire process schematic with inlets and outlets shown, b) the guided pillar route for particles, and c) microscope images of the droplets following the pillar geometry in red and blue food dye. Reproduced with permission from ref (153). Copyright 2011 Royal Society of Chemistry. C) Images from optical microscopy of the channel at different distances along the flow profile where a) no particles flowing and b) two particles seen following the guided rail path, through different coating materials. Reproduced from ref (152). Available under a CC-BY 4.0 license. Copyright 2022 Ziemecka et al. D) The acoustofluidic taSSAW device where a) a 3D illustration of the device comprising of a PDMS microchannel, lithium niobate substrate and deposited IDTs; b) an illustration of the forces and working principles intrinsic to the device operation. Reproduced with permission from ref (157). Copyright 2016 Royal Society of Chemistry. E) Induction of particle migration via the use of magnetic fields for the LbL coating of magnetic particles. Reproduced with permission from ref (15). Copyright 2017 Royal Society of Chemistry.

Practically, DEP has been utilized in LbL by using tilted-angle electrodes to induce directional DEP in microfluidic channels, where particles follow a zigzag motion. When the zig-zagging motion occurs over three parallel, laminar co-flowing streams, layers are assembled on the surface of the particle substrates. By controlling process variabilities like the electrode switching frequency and flow rate, the layering of particles can be controlled. An example of how electrophoresis has been used in this fashion is given in Figure 12A, where 20 μm polystyrene particles and oil droplets were manipulated through a negative polyelectrolyte (PSS), a buffer washing solution, and a rhodamine-123 (Rhod123) positively charged chemical solution. The use of Rhod123 facilitated the use of fluorescence imagery to characterize the particles and hence validate the working principle.^14^

While dielectrophoresis has been used effectively in microchannels to produce multilayered particles, the method is hindered by one particularly important drawback, which is difficult to be applied on nanoparticles or biological matter. This is due to the force on the particle being drastically smaller when the particle size is reduced, an inherent relationship in volume forces, where the volume is proportional to the radius cubed. Conversely, this means that the force required to move or trap the particle is cubically larger also. Furthermore, with the requirement for higher force, a higher power would be necessary, inducing heat. Additionally, while the process is useful in its continuous nature, the throughput of these particles is lowand would not, without large scale microfluidic parallelization, be easily implemented into industrial or clinical applications.

#### 3.6.2 Microfluidic Physical Displacement

Microfluidic physical displacement (MPD) refers to any microfluidic process that uses a physical collision between particles and a channel geometry to cause displacement, often into adjacent laminar flow streams. It is also common to see the term ‘illaring’used for MPD methods, but since the inception of an MPD method that uses railing,^152^ as opposed to pillaring, it is more appropriate to use the parent term MPD when talking about these methods holistically. However, in MPD, pillaring is the most common methodology. This methodology uses physical pillars to guide particles into adjacent flow streams of laminar flow that are enabled by the small channel dimensions, such that the flow streams remain laminar as the particles are guided through an angled path. This process was originally demonstrated with oil droplets of size 45 ± 2 μm, where the specific schematic for this can be seen in Figure 12B.^153^

The most prominent drawbacks of this approach are the inability to fabricate microfluidic chips that can be used to coat particles at the nanoscale. This is often due to scale dissimilarities of the microfluidic channel and the particles, with smaller particles requiring smaller pillars, a difficult feat to overcome with conventional photolithographic processes, where they tend to be useful for feature sizes above 1 μm. However, a similar lithographic procedure, electron beam lithography (EBL), has been shown to be useful in features as small as 20 nm, with high structural control.^154^ The use of EBL to build nanopillars was demonstrated recently in 2020 by Wang et al., for pillars of 150 nm diameter,^155^ but their use in nanoparticle microfluidic coating has not been realized since. Furthermore, although the continuous aspect of this microfluidic approach is desirable, throughput is a prominent drawback. Since the development of the first pillaring system to coat particles, other pillaring devices have been designed, by applying the same principle but in different formats and scales. Notably, research has also gone into coating microparticles, like microbeads. Using parallel walls to contain each of the layering materials, a 6-inlet system was assembled. However, this process did not significantly improve upon the disadvantages of the previous method, including its reluctance to be scaled down to the nanoscale, and hence remains insufficient in being a versatile particle coating system.^156^

An interesting development that followed this established methodology was in particle railing. This methodology uses a vastly different manufacturing procedure and materials. Using only railing geometries at the bottom of the channel, particles were subject to a 7-step layering procedure as proposed by Ziemecka et al.^152^ The approach can eliminate the centrifugal steps, saving processing time and taking around one minute for all layers. Unfortunately, issues were noted in the inability of the rail to contain the particles, with some particles escaping the guiding profile. It was demonstrated on particles of 89 μm in diameter that traveled down the railed pathways into the alternate flow streams. The schematic illustration of this process can be seen in Figure 12C.^152^

#### 3.6.3 Inertial Microfluidics

Inertial microfluidics refer to passive methods that use no physical collisions or external forces to manipulate particles. Instead, they use channel geometries to induce mixing or particle separation. There are many existing methods for particle manipulation on microfluidics; however, there are few methods that have been incorporated into LbL coating. One of the published methods for LbL includes the production of microparticles before their layering commences inside mixing channel geometries. However, the process does not itself constitute LbL in that it incorporates only one layering procedure. Moreover, rinsing is an imperative aspect of the layering procedure, and not one that is accomplished by using this method.^16^

#### 3.6.4 Acoustofluidics

Formed from the incorporation of acoustics in fluid dynamics, acoustofluidics has shown promising signs in LbL coating. Acoustofluidics in LbL coating uses acoustic forces to move particles to or through the relevant coating and washing solutions. Until recently, use of acoustofluidics has focussed on using the primary acoustic radiation force. This force is the primary means to displace particles, often by the use of standing surface acoustic waves (SSAWs), which are a result of two opposing SAWs superimposing upon one another. When the frequencies of these waves are modulated, a standing wave is produced. Most particles will experience an acoustic force directed toward the nodes of these acoustic waves. However, some particles, with particular density and compressibility, relative to their aqueous solution, will experience a force toward the antinodes. It is this acoustophoretic behavior that enables the particles to be controlled. Since these particles can be controlled in this manner, it is possible to hence expose them to the various coating media required for LbL self-assembly. The use of acoustofluidics in LbL coating was originally demonstrated by using SSAWs at an angle nonparallel to the direction of flow. The use of this angled SSAW is termed tilted-angle standing surface acoustic wave (taSSAW). By using taSSAWs, the particles traveling down a microfluidic channel are made to migrate across multiple, adjacent laminar flow streams, passing from a buffer through two reagents and finally being collected in a buffer solution. The process of passing through these laminar flow streams exposes the particle to the necessary coating media, hence layering the particle in the desired regime.^157^ This demonstration is shown in Figure 12D.^157^ Despite this being a promising demonstration of acoustics in LbL, it falls victim to the inherent difficulties in using microfluidic processes in this continuous, particle-by-particle manner: low-throughput. A recent development in 2023 using straight IDTs (nontilted) also sought to use acoustofluidics in particle coating, where SSAWs were utilized to migrate particles over a coating stream; however, this method fails to present a methodology that improves upon the taSSAW methodology when regarding its usefulness in automated industry.^159^ It is worth noting that although the fixed transducer architecture determines the operating frequencies, driving at multiples of these frequencies, or in travelling wave formats, can enable alternatives in the migration and control of particles, an adaptability that is lesser presented in other microfluidic methods, like dielectrophoresis and magnetic fields.

While the limitation of sequential throughput remains prevalent in acoustofluidic approaches to LbL, recent research has shown how traveling surface acoustic waves (TSAWs) have been used to coat a high throughput, nonsequential flow of particles, coating PS particles with PAH.^160^ Cleverly, this development has circumnavigated the restrictions that precise nodal locations have on the number of particles that can be migrated, and hence coated. However, this method shows limited control of the particle microenvironment, is limited to microparticles, and is only demonstrated for one layer, where layering conditions must adhere to flow rates of coating media and sheath flow.

The demonstrated versatility of acoustofluidics in LbL coating presents a compelling opportunity for advancing particle LbL technologies. Acoustofluidic manipulation offers precise control over particle movement and deposition, leading to uniform and tailored coatings on various substrates. Its adaptability across diverse materials and particle sizes makes it an attractive candidate for enhancing the efficiency and scalability of LbL assembly. By leveraging insights from other applications of acoustofluidics beyond LbL, such as microfluidic sorting and multidimensional trapping, researchers can potentially address the current limitations of low sequential throughput and, in the case of TSAW demonstrations, lack of control and size applicability in acoustofluidic LbL technology. Integrating techniques from these fields could lead to innovations in particle LbL assembly, enabling higher throughput while maintaining the versatility and precision that characterize acoustofluidic manipulation. For this reason, consideration will now be given to acoustofluidic demonstrations beyond LbL and a more detailed view of acoustofluidic principles.

The simplest demonstration of acoustic manipulation of particles is in a high-school level experiment of an acoustic levitator. Commonly demonstrated using liquid droplets and polystyrene balls, these objects are seen to float, seemingly magically, between the two transducers. This serves as perfect demonstration of acoustics in object manipulation, of which the principles have been integrated into many novel devices.^161−163^

**Figure 13:**
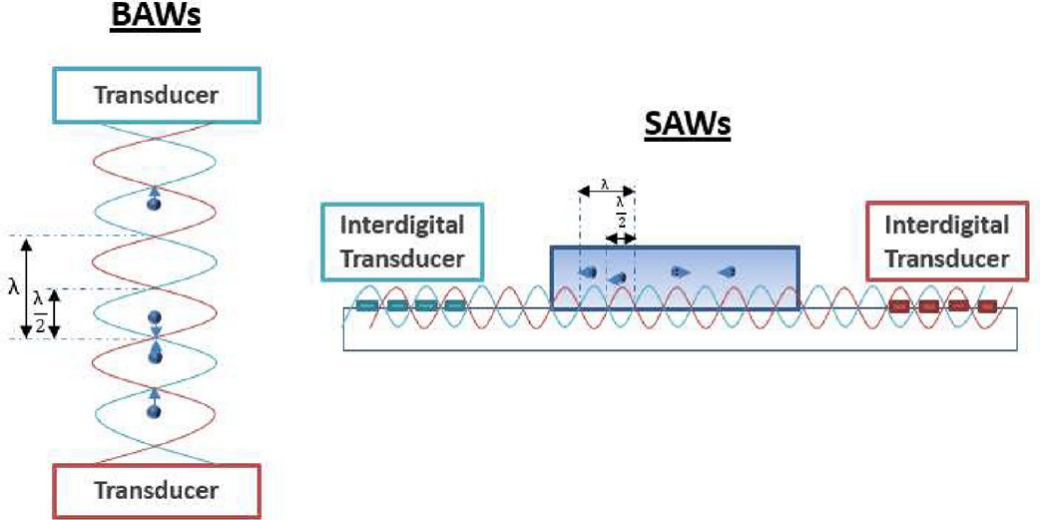
An illustration of an acoustic levitator using BAWs and the comparison of this to a device that traps particles using SAWs, where the wavelength is given as λ, and particles can be seen experiencing a force toward the nodal positions of the standing waves.

Acoustic levitation belongs to a family of acoustic particle manipulation applications, which, when mentioned generically, are often referred to as acoustic tweezers or acoustic tweezering. Acoustic tweezers refer to the acoustophoretic motion of media due to acoustic forces.^164^ Acoustic tweezers can come in various types, of which each use a different construction of acoustic sources to produce the tweezering effect; standing-wave tweezers, traveling wave tweezers and streaming tweezers.^165^ For now, the focus will remain on standing wave acoustic tweezers, although the alternatives will be mentioned to exemplify their use in the field. To facilitate standing acoustic waves, one transducer and a reflector or two opposing transducers are required. In both cases there are two acoustic waves propagating in the same plane, in opposite directions. As seen in earlier demonstrations in LbL, by selecting the correct driving frequencies, this interference can be tailored to create a standing wave. The physical phenomena that control the behavior of particles within this acoustic wave field mean that a particle, depending on its density and compressibility and that of its surrounding media, will migrate to the nodes of antinodes, points of minimum and maximum amplitude, respectively, of the wave.^166^ An example of an acoustic levitator, with a standing acoustic wave formed between a transducer and reflector, using bulk acoustic waves (BAWs) or SAWs can be seen in Figure 13.

In standing wave acoustics there are two main categories of acoustic devices, based on their propagating and originating constraints. These are BAW and SAW devices. A comparison of these two types can be seen in Figure 13. Both mechanisms have been used in a variety of acoustophoretic applications.^167,168^ However, regarding the focus of this review on particles including nanoparticles, SAWs are particularly attractive, due to significant benefits over the use of BAWs that include better control of excitation frequencies in a wider range resulting in higher precision (useful for nanoparticle manipulation), simplicity in design and manufacture since they do not require highly reflective acoustic boundaries, meaning that PDMS can be used in accordance with standard microfluidic soft-lithography protocols,^165^ compactness, and less heat being generated at high power due to the displacement field being localized to the surface of the medium.^169^ While BAWs can offer promise in their use in higher throughput, it is hypothesized that with novel design SAW devices can achieve similar levels, suitable for industrial and clinical adoption.^165^

It is also worth mentioning the existence of acoustic devices that use traveling waves, rather than standing waves. While still regarded as tweezers since they manipulate particle matter, these devices are principally different. As the name suggests, the acoustics waves are traveling waves, hence they do not use the interference and superimposition of standing wave tweezers. However, these systems either require many transducers, inheriting complexity and expense,^170^ in the case of active traveling waves transducers, or inherent complex simulation and calculation requirements in the case of passive traveling wave devices.^171^ Both have limited use in the nanoparticle range.

To demonstrate the promise of acoustics in particle manipulation strategies it is important to provide a fuller picture of the number of ways in which acoustofluidics has been applied beyond LbL coating. This section will consider some of the most common technologies that integrate microfluidics and acoustics, such as cell sorting, and one-dimensional and multidimensional traps for singular and multiple particles.

**Figure 14:**
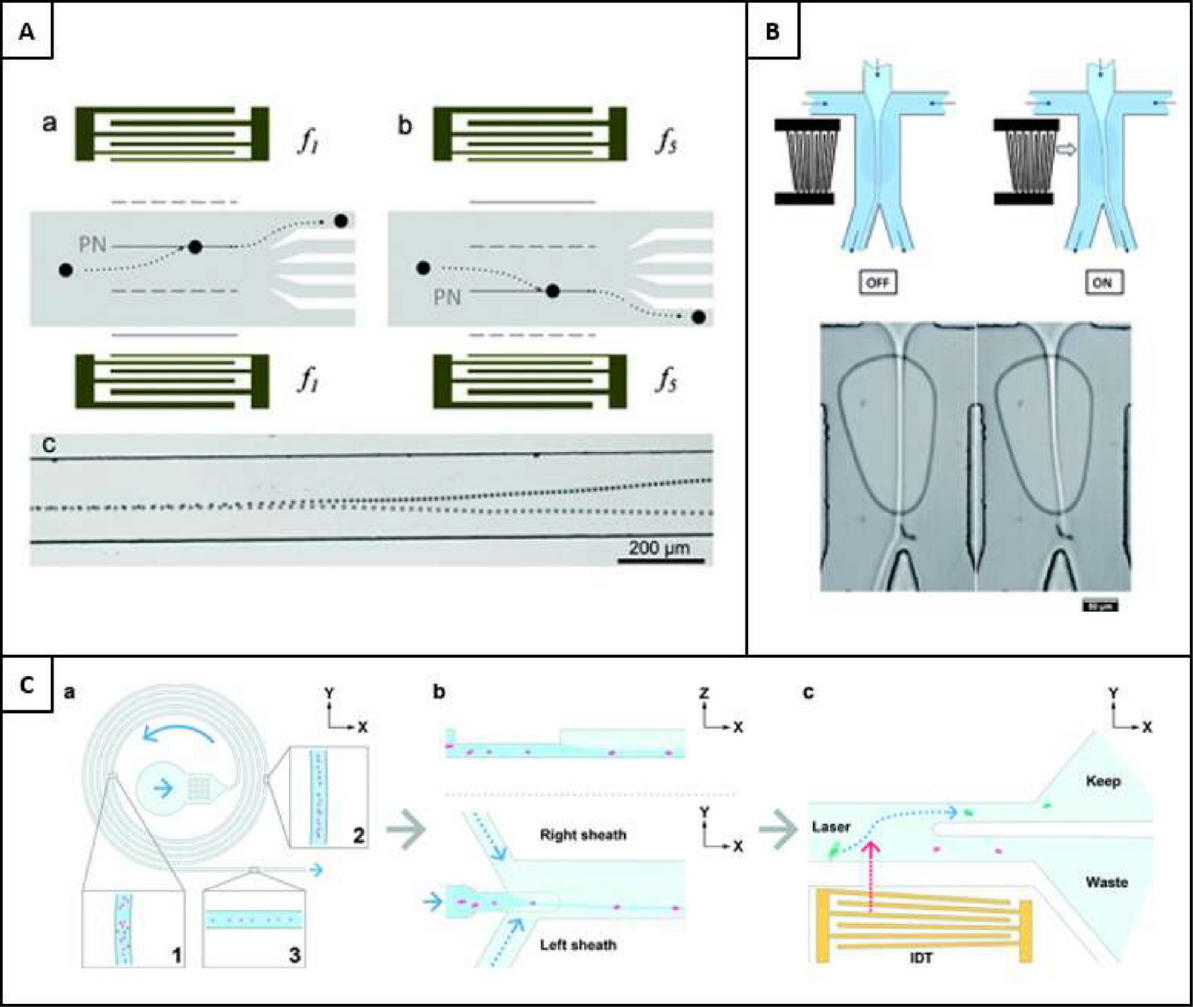
A) Standing surface acoustic waves being used to sort particles where frequency modulation changes the location of the pressure node, from a) frequency f1 and b) frequency f2 and c) corresponding particle deflections. Reproduced with permission from ref (175). Copyright 2012 Royal Society of Chemistry. B) Acoustic streaming being used to deflect a stream of particles from one outlet to another. Reproduced with permission from ref (174). Copyright 2010 Royal Society of Chemistry. C) A process by which a) particles are initially focused into a single file via the spiral channel, b) acceleration and further alignment by use of two sheath flows, and c) laser interrogation of fluorescence and deflection by a traveling wave. Reproduced with permission from ref (176). Copyright 2019 Royal Society of Chemistry.

Owing to the versatility, high biocompatibility, and simple design acoustofluidic particle sorters have retained a focus in recent years. Acoustofluidic techniques in cell sorting involve the separating of particles based on their physical and chemical properties. Devices of this type have been demonstrated with each of the acoustic wave forms, standing waves,^172^ traveling waves,^173^ and acoustic streaming,^174^ where illustrations of these can be seen in Figure 14.^174−176^ These devices demonstrate the usefulness of acoustofluidics in particle manipulation, for a range of particles sizes, from micro- to nano- particles.

**Figure 15:**
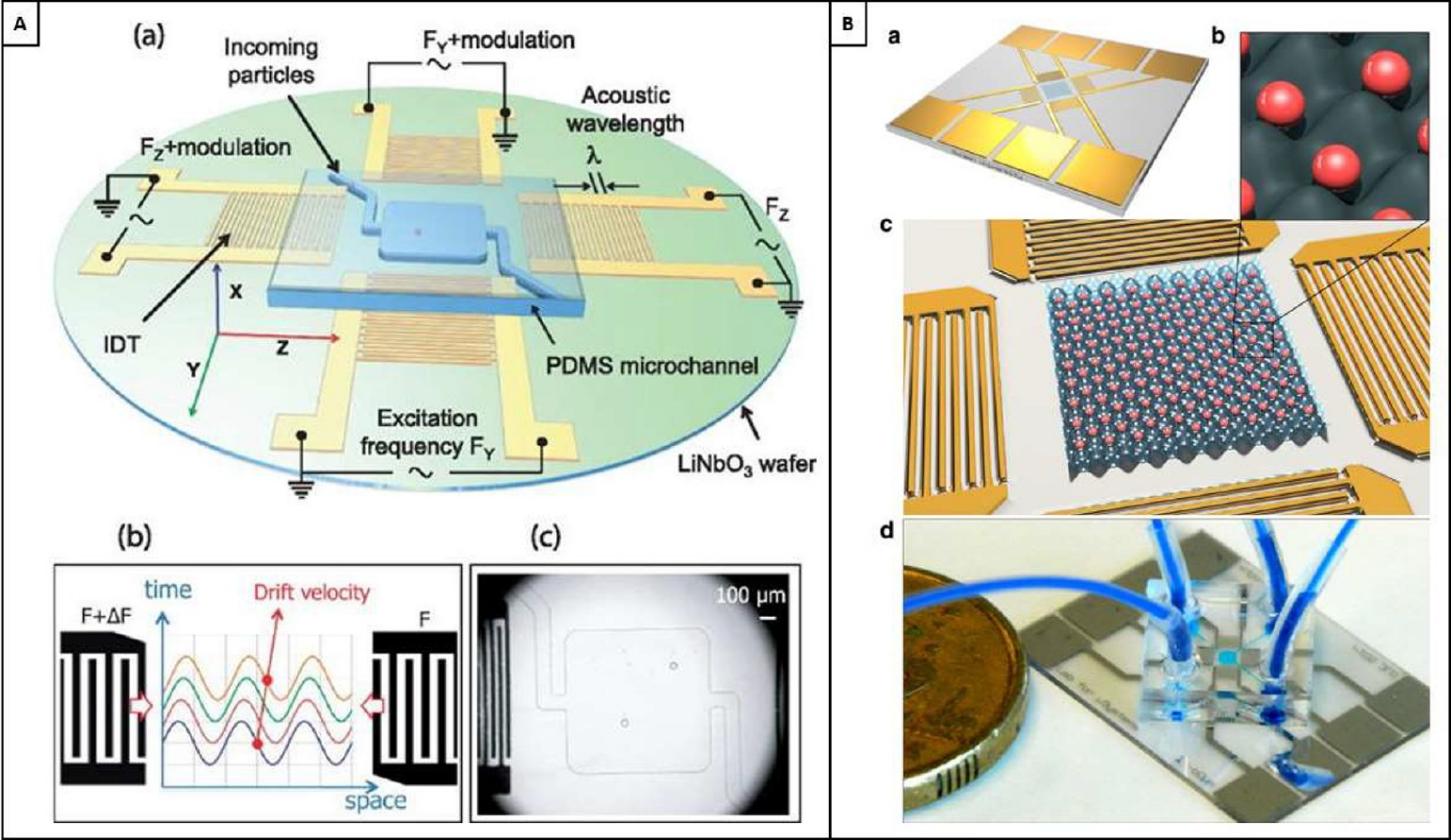
A) A single particle manipulator that uses the principle of acoustic tweezers to trap and maneuver particles via frequency modulation of opposing transducers, where a) scheme of the acoustic device geometry, b) principle of frequency modulation to move particles, c) image of two oil droplets trapped in the chamber. Reproduced with permission from ref (180). Copyright 2012 AIP Publishing. B) An acoustic trap that captures one particle per well, where a) a scheme of a OCPW trap with chamber and IDTs, b) and c) trapping of individual particles, and d) channel visualization with blue dye. Reproduced from ref (178). Available under a CC-BY 4.0 license. Copyright 2015 Collins et al.

While cell sorters are usually considered with just one dimension of acoustic radiation, there also exist several devices in the literature that use acoustofluidics in multiple dimensions. These devices trap and manipulate singular^177^ or many^178^ particles and cells. By incorporating multiple acoustic wave sources, often orthogonally, the acoustic field pattern of nodes and antinodes appears as a square array of minima and maxima amplitude, respectively. Commonly, particles are held at one of these nodes, or in clusters of particles across multiple nodes. This process is often referred to as acoustic tweezering in 2D, where 3D particle trapping has also been demonstrated.^179^ These devices use SSAWs to create the desired pressure field. In Figure 15,^178,180^ single and multiple particle traps are illustrated, where the multiple particle trap is a one cell per acoustic well (OCPW) trap. These devices have been demonstrated on a range of particle sizes, from microparticles to nanoparticles,where issues in the manipulation of sub-100nm particles have been addressed by coupling the acoustic forces with electrical amplification, in acoustoelectric particle manipulation.^181^

Referring back to existing acoustofluidic LbL technologies and their previous applications, a significant disadvantage is the limited throughput of the process. However, the demonstrations presented here for multiple particle trapping show promise for improving upon the sequential processing that limits current methodologies, if such methods could be utilized.

While recognizing acoustic tweezering for its general particle manipulation benefits, it is useful to compare it to alternative tweezering methods, such that acoustofluidics appears not only as a most favorable means of particle-LbL, but of all other tweezering techniques also, including optical tweezers,^182^ magnetic tweezers,^183^ optoelectronic tweezers,^184^ plasmonic tweezers,^185^ electrokinetic tweezers,^186^ and hydrodynamic tweezers.^187^ Although these other methods have not been incorporated into LbL, they remain important methods for particle manipulation,where a usefulness in particle manipulation can often highlight an eligibility for use in LbL coating. However, despite the development of these technologies, acoustic methods retain promise when compared to its tweezering peers. In contrast to the others, acoustic tweezers are effective for particle sizes that are commonly found in LbL demonstrations, from 100 nm to 10 mm, where the prior makes it suitable for use in biomedicine and pharmaceuticals. One of the reasons for this is that the input power required to control particles is vastly less than other methods, for example while optical tweezers require input power of 10^6^ to 10^7^ W/cm^2^, acoustic tweezers require input power of 10^–2^ to 10 W/cm^2^.^165^ Additionally, acoustofluidics continues to be attractive in that, unlike other methods, acoustic tweezers do not require any labeling of particles. For these reasons, as well as its already evidenced use in LbL, acoustic tweezers, and more generally acoustofluidics, shows promise in its application in particle coating.

#### 3.6.5 Magnetic Microfluidics

Similar to other zig-zagging microfluidic approaches, the principle behind this technology follows the same microfluidic behavior seen in acoustofluidics. The process of magnetic microfluidics employs a microchannel with multiple laminar flow streams, introducing particles in one stream and applying a magnetic force with a component perpendicular to the flow direction. This induces the migration of particles through various coating media. However, this process is severely limited to coating only magnetic particles.^15^ Due to this limitation, the process will not be mentioned in detail, but should be acknowledged as an existing technology in this field. The schematic for this device is shown in Figure 12E.^15^ Recent publication in field of magnetic microfluidics has reproduced this behavior but for four layers.^158^

### 3.7 Filtration

Using membranes to separate particles and their suspending media is a useful technique in the particle coating realm. While the size of the particles being coated presented problems for the first filtration methods along with caking of filter membranes, two poignant developments have sought to overcome the issues first seen in filtration-based particle-LbL.

#### 3.7.1 Membrane Filtration

**Figure 16:**
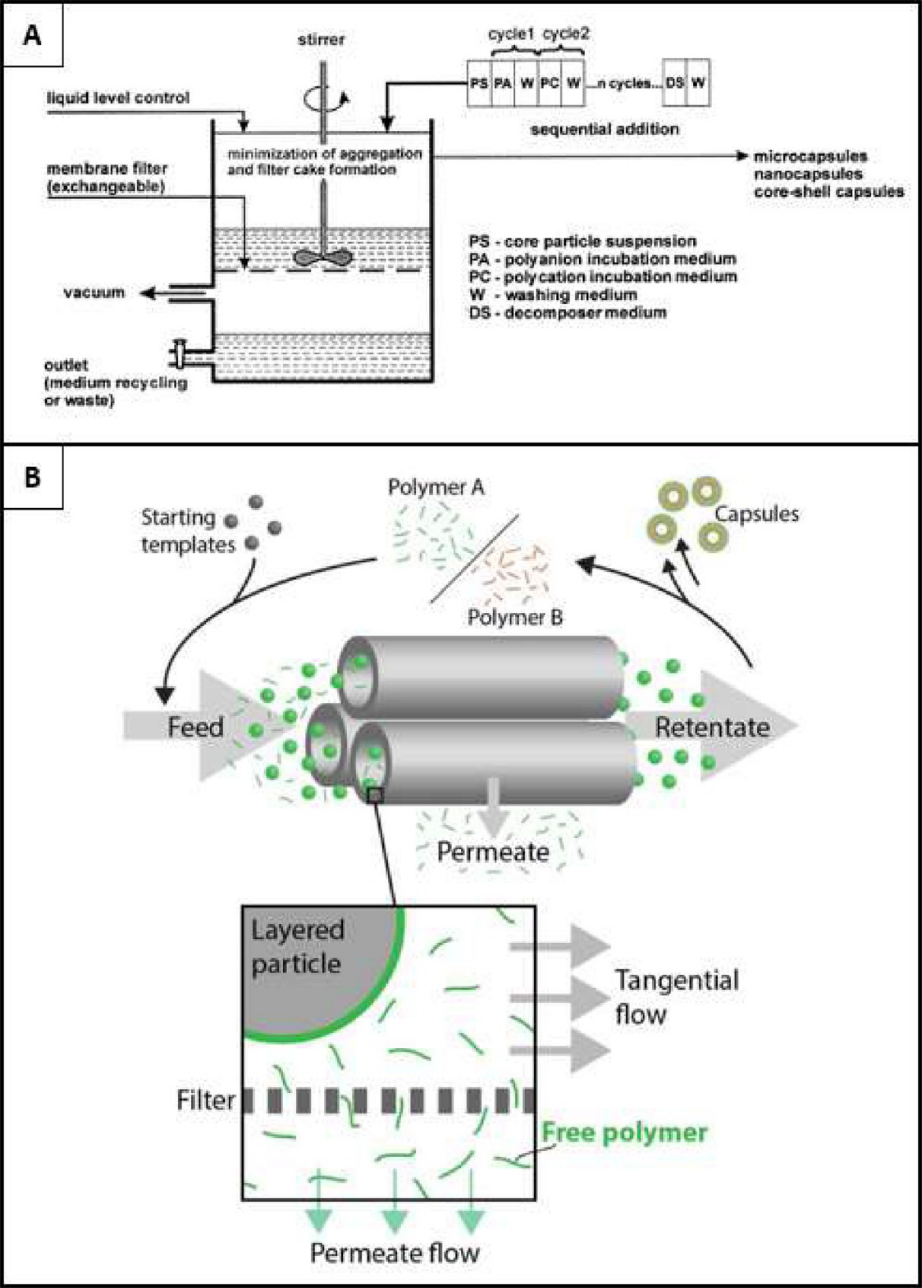
A) Membrane filtration for microencapsulation and microcapsule formation via LbL coating. Reproduced from ref (188). Copyright 1999 American Chemical Society. B) The process of TFF LbL developed in 2015, where starting templates are exposed to a coating media (polymer A/B), the permeate is then removed by tangential filtration, and the particles are then exposed to the next coating media (polymer B/A) to form capsules or coated particles. Reproduced from ref (189). Copyright 2015 American Chemical Society.

Filtration-based LbL was first demonstrated in 1999 with the coating of polystyrene sulfate latex and soluble melamine formaldehyde resin latex particle substrates, as well as decomposable glutaraldehyde fixed human red blood cells. The demonstration used common coating materials, PAH, PSS, poly(diallyldimethylammonium chloride) (PDADMAC), chitosan, and chondroitin sulfate.^188^ The methodology uses vacuum filtration, pressure filtration, and filtration without pressure, where the filtration takes place through a membrane. The membranes must be chosen based on the charge of the absorbing polyelectrolyte, to prevent clogging, and the pore size to account for variations in the desired particles that are being coated. A schematic for this membrane filtration process is given in Figure 16A.^188^ This filtration method was invented early on in the development of LbL technologies, where only two methods had been demonstrated previously. These methods were centrifugation and the addition of highly specific and accurate quantities of the coating materials corresponding to monolayer coverage.^8^ This method of filtration produced positive results compared to the inadequacies of the previous methods. However, it suffered from some major disadvantages, like lack of automation and applicability only in the micron range. Additionally, significant disadvantages were also seen in the behaviour of the membranes, including the modularity of the membrane filters and filter clogging. It is these issues that subsequent filtration methods have sought to resolve.

#### 3.7.2 Tangential Flow Filtration

Tangential flow filtration (TFF) is a methodology utilized by chemists and engineers in a range of applications, including being demonstrated for the first time in the field of LbL by Björnmalm et al., in 2015.^189^ The methodology takes particles or templates and exposes them to one of the two coating solutions. While exposed, the particles undergo the self-assembly inherent to LbL. Then, particles are filtered from their surrounding media and the permeate is removed. The process is then repeated with the second coating media, so on and so forth. The schematic in Figure 16B demonstrates the process.^189^ The method is regarded as well-controlled, integrated, and automatable; however, the automation is yet to be practically demonstrated. Whilst the process is shown to be applicable on submicron particle sizes, shows great reproducibility, and can perform additional LbL steps like core dissolution, the process principally suffers from a yield per layer like that of centrifugation. This is a major drawback of the centrifugation LbL protocols and is hence a major drawback for TFF LbL. In the same journal that initially published this method, Björnmalm et al. also reported that the yield dropped by an order of magnitude (10^10^ to 10^9^) for the number of particles output after 8 layers, following a similar trajectory of the centrifugation. This was evidenced by flow cytometry. It was determined after investigation that the issue lied in the filter. Particles were becoming adsorbed to the surface and in the network of porous geometry. While consideration is given to adaptations to the filters that may offer a higher yield, this is not demonstrated.^189^

## 4 Applications

The applications for LbL particles have been rapidly developed since their inception, largely due to the versatility of the self-assembly method. The wide range of eligible materials means that many application areas have been researched. This section will report on the main applications of LbL particles, predominantly drug delivery, but will also cover use in theranostics,food applications and water treatment. Alternative applications can also be envisioned, like in cosmetics; however, these are not currently seen in literature.

### 4.1 Pharma/Drug Delivery

LbL particles have demonstrated use in several fields in disease therapies, due to their ability to target specific sites within the body and tailor the interaction of the drugs with their environment. While the literature of LbL particles in drug delivery can engender a bewilderment due to the number of particle types, the scope of this section will consider two forms of LbL particles, core–shell nanoparticles (CSNs), as well as hollow capsules, like polyelectrolyte multilayer hollow capsules (PMCs). These LbL particles have been demonstrated on several diseases, of which some are explained in the following subsections. It is worth noting that these examples are nonexhaustive and that many other examples exist in the literature beyond that mentioned herein.

#### 4.1.1 Immunotherapy

The generation of strong T-cell responses and robust antibody-mediated immune responses from vaccinations has been a major challenge for the medical field.^190^ Due again to the versatility, LbL has been gaining traction as a methodology for solving this challenge. LbL particle vaccines can transport systemically toxic cargo, while reapplying the inherent ability to specifically target sites with active outer layers. The use of LbL in immunotherapy has been demonstrated thoroughly over the last two decades. Earlier development included using LbL to stabilize and coat antigen-salt precipitates^191^ and polyelectrolyte-antigen complexes.^192^ More recently, direct incorporation of antigen peptides into LbL films on nano range particles has produced interesting results. This has been implemented recently in the production of a LbL nanoparticle vaccine. Using AuNPs, functionalized through the LbL method with sequential deposition of the anionic adjuvant polyinosinic-polycytidylic acid (polyIC) and a cationic SIINFEKL peptide antigen, strong T-cell proliferation and dendritic cell maturation was induced successfully.^193^ These demonstrations are encouraging for the use of LbL particle vaccines, where the use of LbL vaccines will be further fueled by other parallel developments in immunotherapies, like amphiphile-based immunotherapy.^194^

#### 4.1.2 Gene Therapy

Gene therapies are considered to be medicinal products that contain an active substance that consists of a recombinant nucleic acid, present in order to regulate, repair, replace, add to, or delete a genetic sequence, with the therapeutic, prophylactic, or diagnostic effect being a result of said nucleic acid.^195^ In short, it is the introduction of normal genes to cells, replacing missing or defective native genes. The mechanism for transporting and targeting is vitally important to the effectiveness of the therapy. These difficulties have been addressed by using LbL films since the 1990s where DNA was incorporated into planar films.^196^ More relevantly, this process was demonstrated on colloidal substrates in the early 2000s^197^ as well as in the generation of DNA-loaded LbL microcapsules.^198^ Research has shown how LbL particles are able to increase the efficiency of gene delivery uptake into cells^199^ and cytosolic release of nucleic acid cargo,^200^ as well as producing desirable subcellular trafficking via endosomal escape.^201^

Demonstrations of dual loaded LbL nanoparticles using gene therapy also exist. In 2016, AuNPs were used as cores for biodegradable polymeric coatings that simultaneously delivered DNA and siRNA. This dual approach smoothly delivered DNA to the cells and saw that the siRNA gene silencing effect was better than that of commercially available transfection reagents.^202^ Dual gene formulations of LbL nanoparticles have also used a combination of two different types of plasmid DNA. The tuning of the assembly order of these layers was shown to affect the DNA expression time,^203^ supporting the usefulness of LbL as a versatile method of gene therapy.

#### 4.1.3 Chemotherapy

The treatment of cancer has been, and remains, a prominent challenge in today’s society, with the disease’s complex pathological process making it troublesome to comprehensively solve. Chemotherapy is a popular solution; however, it is not without a plethora of challenges itself. Chemotherapy’s problems include a lack of specificity and cytotoxicity,^204^ meaning it still remains the leading cause of death in patients globally.^205^ However, looking at these problems and considering its success in the aforementioned sections, it is easy to see that LbL beholds great promise in the addressing of these issues.

Nanodrug delivery systems (NDDSs) is a term commonly referred to when talking about particles carrying a drug payload on the nanoscale, in which the nanoscale is particularly useful for anticancer treatment. There exist many names for these drug delivery systems, another name commonly used is polymeric nanoparticles. The benefits of these particles, regardless of their name, have been summarized in literature.^206^ NDDSs are commonly manufactured via nanoprecipitation, solvent evaporation, and in situ polymerization. Unfortunately, these methods present a lack of versatility, a versatility that is required when combatting the issues in chemotherapeutic drug delivery, like specificity and toxicity. LbL presents a valuable method for the development of NDDSs that combat these challenges. The LbL methodology can produce homogeneous nanoparticles, as well as heterogeneous nanoparticles with complex multilayer structures, controllable thicknesses, surface charges, morphologies,^207^ and, depending on the interlayer bonding mechanism utilized, can tailor the thermal and mechanical properties, while loading with hydrophobic and hydrophilic drugs.^208^

**Figure 17:**
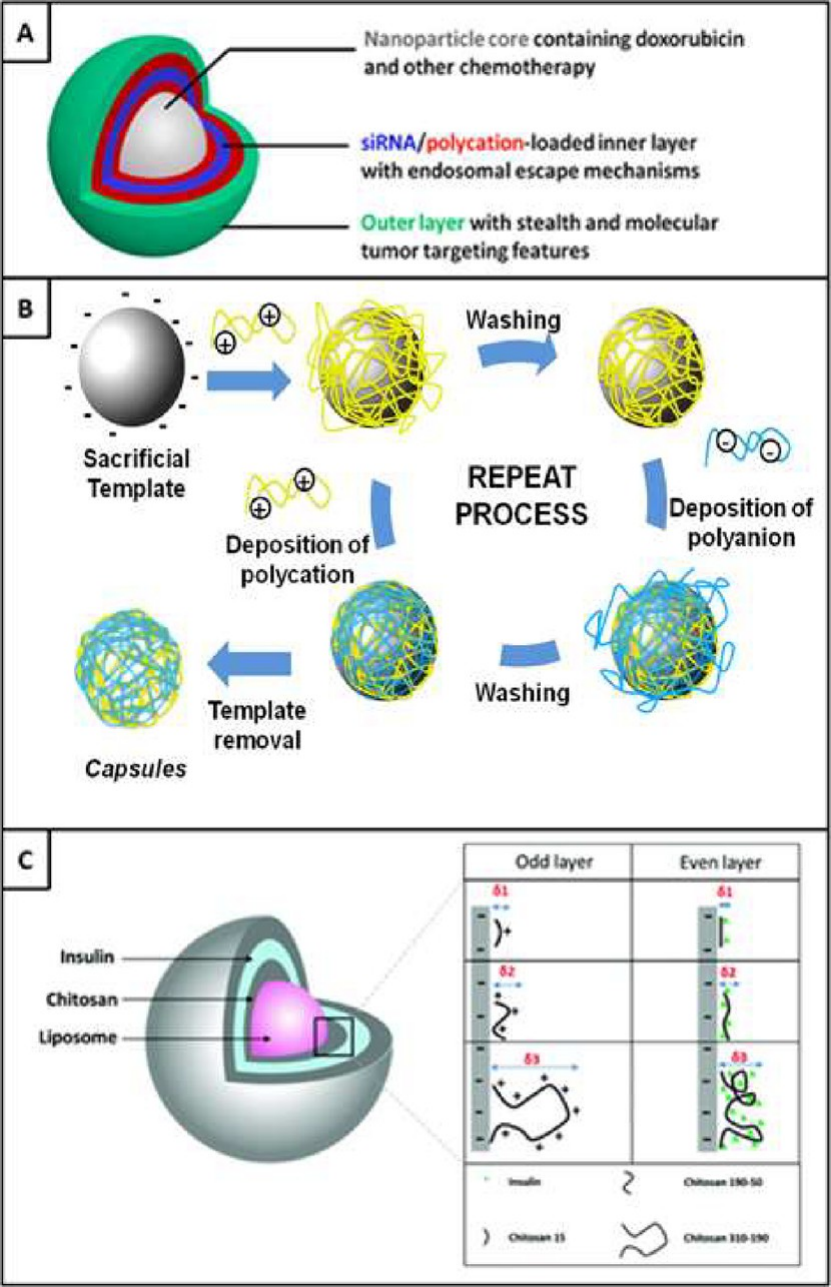
A) Schematic of a modular combination drug delivery platform based on LbL nanoparticles. Reproduced from ref (199). Copyright 2013 American Chemical Society. B) The formation process of polymeric capsules from templates via the use electrostatic LbL. Reproduced with permission from ref (214). Copyright 2021 IOP Publishing. C) A schematic of a LbL drug particle, loaded with insulin in chitosan polymeric layers. Reproduced with permission from ref (216). Copyright 2020, Royal Chemical Society.

Specific applications of these LbL polymeric nanoparticles in cancer treatment have been demonstrated in treatment of breast cancer^199,209^ and ovarian cancer.^210,211^ In this application it is quite common to see a coupled approach in the treatment, combining siRNA and chemotherapeutic methods, a combination that is facilitated by the versatility of LbL. This is useful because more complex cancer cells can behold an ability to repel the chemotherapeutic nanoparticle. The use of siRNA can disable this gene, allowing for successful uptake of the chemotherapy into the cancer cell. Recent studies in to identifying the optimal surface chemistry for a nanoparticle being used to treat ovarian cancer cells have also been conducted, showing that poly-l-aspartate, poly-l-glutamate, and hyaluronate coated particles showed a particular affinity to ovarian cancer cells, demonstrating the effectiveness of LbL as a method for tuning the tumor targeting ability of nanoparticles.^211^ An example of how LbL nanoparticles, consisting of a chemotherapeutic drug-loaded core, siRNA complex multilayer structure, and tumor targeting outer shell can be seen in Figure 17A.^199^

The combination of gene therapy and chemotherapy is not the only example of how LbL nanoparticles have been structured to incorporate chemotherapeutic drugs. Formulations of dual loaded anticancer LbL particles have been demonstrated in literature, too. A combination of doxorubicin and mitoxantrone was used in the assembly of nanoliposomes for the codelivery of chemotherapeutic agents. This was enabled by the sequential deposition of poly-l-lysine (PLL) and poly(ethylene glycol)-*block*-poly(l-aspartic acid) (PEG-*b*-PLD). The results of this study showed a significant reduction in the clearance rate of the two drugs and a prolonged circulation time in the body of two male rats.^212^

#### 4.1.4 Antibacterials

Antibiotics pose a troublesome prospect for medicine, both in the engenderment of antibiotic resistance in the population, but also in the toxicity of high concentrations in blood. For these reasons, among others, there is a desire for alternatives. The field of LbL particles holds some promise in this area. Some polyelectrolytes, with inherent bactericidal components, exist as quaternary ammonium or phosphonium groups. These charged polymers and the negatively charged bacterial cell walls can interact, resulting in the degradation of the bacterial cell envelope. Where previously hollow capsules were noted to exist primarily in the nano range, here an example of the formation and usefulness of microcapsules can be seen. Using CaCO_3_ particles as templates, multilayered coatings have been created, following this the template is removed leaving a polymeric microcapsule. Used commonly in drug delivery due to its biocompatibility, chitosan was demonstrated as a useful material in this application, performing the desired antibacterial interaction with the bacterial cell walls, with a dextran derivative as the complementary layering material. While demonstrating that this chitosan-dextran derivative LbL particle served as an alternative to antibiotics, it was the mechanical properties of the particle that were of focus. The resulting flexibility, compared to antibiotic solid CaCO_3_ particles, facilitated an easier interaction with bacterial walls, inhibiting bacterial growth.^213^ Further and more recent studies have supported the results, applying, and studying the use of sacrificial CaCO_3_ templates to form polymeric capsules with antibacterial properties, interacting with antibiotic resistant kanamycin-resistant *Escherichia coli*. Figure 17B^214^ illustrates the fabrication of capsules through LbL of generic sacrificial templates.

Agreeing with the necessity to find methods to limit and finetune the quantity of antibiotics used in a fight against the generation of antibiotic resistance, other studies have used the LbL approach. Although not strictly LbL, one study created a single monolayer on gold nanoparticles (AuNPs) consisting of Coliston, a last line of defense antibiotic with low antibiotic resistance but undesirable side effects. LbL is used as a method to limit the dosage of Coliston due to these side effects. The argument for this being a LbL procedure is in the use of an electrostatic bonding mechanism between the Coliston and the AuNP. Conversely, LbL enthusiasts may argue that a single monolayer is insufficient to be labeling this as a LbL process. However, regardless of the number of layers, this study demonstrated the effectiveness of the electrostatic self-assembly process to precisely deliver dosages of Coliston. Furthermore, the LbL process demonstrated here would allow for further incorporation of additional layers if desired.^215^

#### 4.1.5 Insulin Delivery

The body’s digestive system is thwart with barriers that prevent the sufficient migration of drugs in the body. In the case of oral delivery of insulin in diabetic patients this is no different. Orally delivered insulin must cross the intestinal epithelial cell barrier safely and reach the blood with therapeutic levels of bioactive insulin to be effective. While efforts have been made to design drug formulations that achieve this, the gastrointestinal (GI) environment makes this difficult, owing largely to the extreme variations in local acidity. Multilayered insulin delivery systems, belonging to the CSN family with a liposome core, manufactured with the LbL self-assembly process, have performed well in achieving this desired transport. The LbL process can be used to load insulin into the shell layers, as well as the intermittent counterionic polyelectrolytic layers, producing a high insulin loading capacity that delivers sustained release kinetics, due to the defoliation of layers overtime. This process, demonstrated on a nanoliposome core, outperformed their noncoated nanoliposome counterparts, delivering higher insulin loading, better protection to the GI environment, and higher penetration of the intestinal epithelium with retention of bioactivity. Figure 17C shows the schematic of the insulin loaded LbL formulation.^216^ Furthermore, the assembly inherent to LbL shows great promise in that the quantity of insulin can be controlled by varying the number of layers in the shell formulation, as well as versatility in the core size.

#### 4.1.6 Osteotropics

While insulin delivery serves as a demonstration of the use of LbL particles in the transport of drugs through the human body, demonstrations of LbL particles in osteotropics focuses on the targeting abilities of LbL coated CSNs. Many bone-based diseases cause undesirable disturbances to the osteoblast and osteoclast mediation of bone deposition and resorption. An example of this can be seen in osteosarcoma. Therapies that address this abnormal behavior are suboptimal and have low efficacy. These issues have been addressed by LbL particles, namely, LbL of liposomes. While the core is loaded with chemotherapeutic agent doxorubicin, the shells are designed to target specific locations, local to the osteosarcoma. The trafficking of LbL particles to ectopically induced 143B osteosarcoma xenografts in mice was evidenced with *in vivo* fluorescence imagery, where the doxorubicin payload was then released. This successfully demonstrated the usefulness of LbL liposomes in the targeting of bone disease.^217^

#### 4.1.7 Cell Encapsulation

A final application of LbL in the drug-delivery sector is in cell encapsulation. Cell encapsulation, sometimes referred to as cell modification, has only been mentioned briefly so far in this review; however, developments in cell encapsulation continue to impact drug delivery, as well as in fields of cellular biosensors^218^ and catalysts,^219^ and hence should be considered as a field of application by its own merit. As such, the field is extensive and well-reviewed^220^ and many of the applications sit outside of the scope of this review. The use of LbL for encapsulation is beneficial due to the time and economic efficiency it possesses over alternative methods.^17^ In 2000, the original study of LbL for cell encapsulation applied a PAH and PSS regime on erythrocytes.^221^ Since this demonstration, cell encapsulation via LbL has been shown to be useful for many useful complexes. The building blocks of these complexes include polycations such as polyethylenimine (PEI),^222^ poly(dimethyldiallylammonium chloride) (PDDA)^223^ for nonmammalian cells, and cationic chitosan,^224^ gelatin,^225^ cellulose,^226^ poly-l-lysine (PLL),^227^ and polyamidoamine^228^ for mammalian cells. Providing the opposing charge, polyanionic materials have included alginate,^229^ hyaluronic acid (HA),^230^ and PSS.^231^

### 4.2 Theranostics

**Figure 18:**
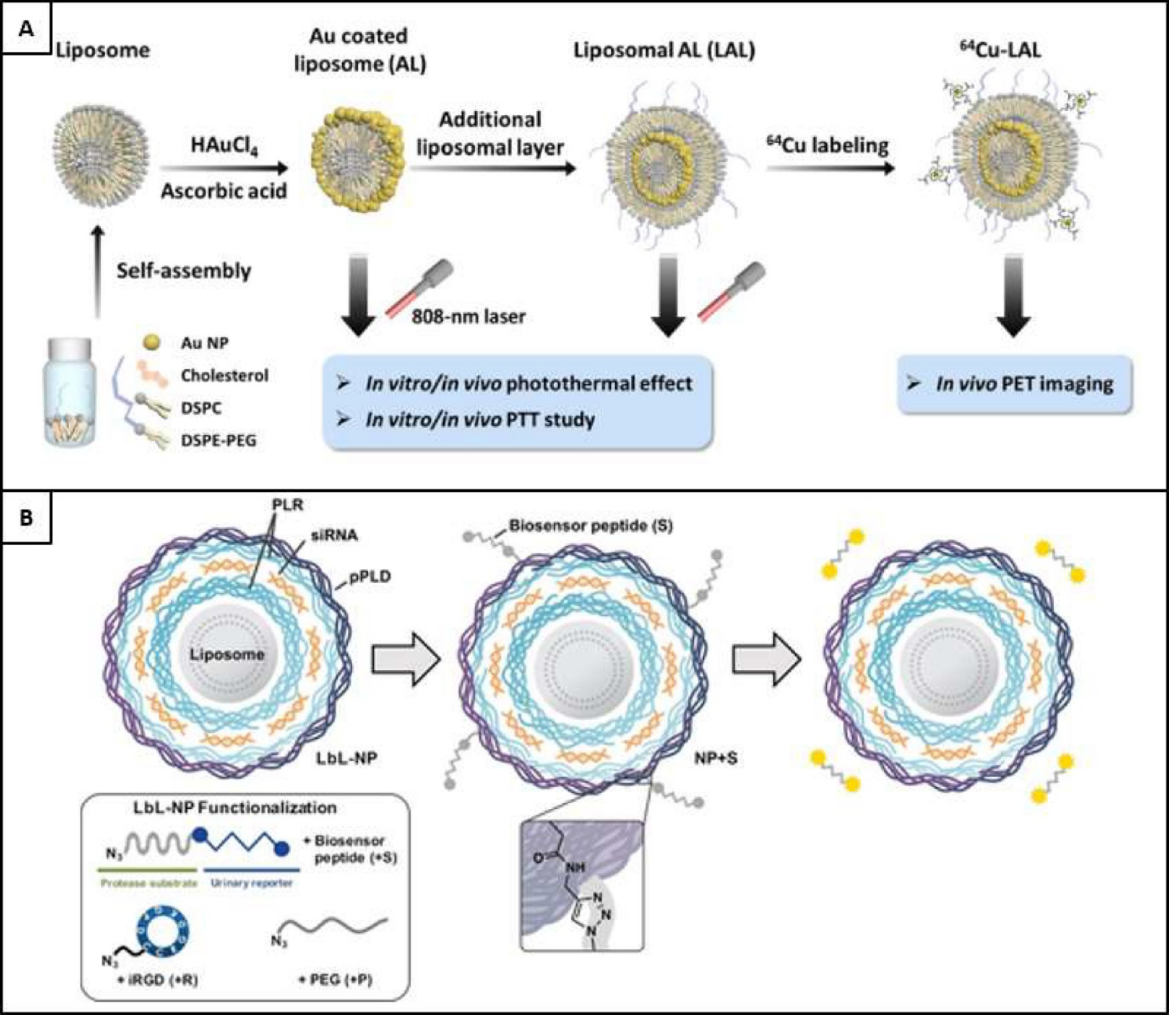
A) A schematic showing the LbL process for producing Cu labeled liposomal gold liposome (AL) particles and their intermittent usefulness under 808 nm laser activation or PET imagery. Reproduced from ref (233). Available under a CC-BY 4.0 license. Copyright 2021 Jeon et al. B) A schematic showing the LbL nanoparticle construction and biosensing peptide separation for urinary-based diagnostics, where PLR = poly-l-arginine, pPLD = propargyl-modified poly-l-aspartic acid. Reproduced with permission from ref (234). Copyright 2019 WILEY-VCH Verlag GmbH & Co. KGaA Weinheim.

Diagnostic applications for nanoparticles have been evidenced widely in research. Naturally, this application has also been shown for LbL particles. However, where standard nanoparticles are only useful for diagnostics, LbL particles can integrate the delivery of a therapy also, leading to their more recent, extensive research in the field of theranostics.^232^ This is unsurprising since LbL has been shown to be a methodological protocol, where one challenge is met with the application of one layer, and the next challenge is met with another layer. Here, the challenge is to have one layer produce a therapeutic effect and the other is to facilitate diagnostics. LbL nanoparticle theranostics is hence implementable with the versatile range of input materials inherent to the LbL procedure, like chemotherapies and gene therapies, as well as enabling different diagnostic methods, like imaging, tracing,^233^ and urinary- based diagnostics.^234^ Diagnosis through positron emission tomography (PET) has been well demonstrated in the use of gold-liposomes, where one of the layers consists of AuNPs and the outer surface is a second liposomal layer labeled with copper (^64^Cu). The AuNPs facilitate the therapeutic effect via photothermal effects under irradiation under 805nm electromagnetic radiation and the copper enables the radiolabeling for imaging via positron emission tomography (PET), schematically shown in Figure 18A.^233^ Similar studies in the visualization-based diagnosis of ovarian cancer have also been conducted, using LbL to incorporate second near-infrared (NIR-II) fluorescence imagery and drug delivery, resulting in sustained accumulation of NPs in diseased sites, characterized through the fluorescence based imagery, enabled by NIR-II properties of the LbL nanoparticle layers.^235^ Additionally, urinary-based diagnosis methods have used LbL nanoparticles. These particles work by using biosensing peptides that are activated by particular proteases that occur in the cancer microenvironment. When subject to said proteases, a synthetic biomarker is released that can later be detected in urine. This mechanism has been integrated into LbL particles in addition to gene silencing siRNA, and a number of polymer monolayers, as illustrated in Figure 18B.^234^

### 4.3 Food Industry

Food preservation depends on a variety of factors like formulation, processing, packaging, and storage conditions. The incorporation of LbL microparticles is hence included in formulation. To extend the shelf life of food items it is desirable to suppress bacterial growth and minimize lipid oxidation. Preservation of food is attained by either adding additives that release over time, or additives that are activated by a change in environment, a change that is brought about by the aging of the food itself. Due to the continuous supply of actives, the use of these additives is the most advanced form of food preservation, prolonging the shelf life without compromising flavor, taste, or nutrients.^236^ A demonstration of the usefulness of LbL microparticles as food preservative additives has been shown by producing a double-layered particle, with a pH-responsive antibacterial shell and a nonresponsive antioxidant core, of benzoic acid in Ac-dextran (acetalated-dextran) and tocopherol in amorphous PLGA, respectively. These material and active component combinations were selected to enable the faster release of antibacterials, once triggered by low pH, and to facilitate an active release of antioxidants from the core at a desirable rate.^237^ This demonstration illustrated successfully how the LbL approach could be used in the application of food preservation by careful selection of the coating materials. Furthermore, LbL particles have also been evidenced to be useful in is in food fortification. A lack of dietary fiber (DF) has been shown to be a prominent issue in societal health,^238^ including both soluble dietary fiber (SDF) and insoluble dietary fiber (IDF). IDF is obtainable via okara powder, a type of soy pulp that arises as a byproduct of soy milk and tofu production. Okara insoluble dietary fiber (O-IDF) can be used as a food additive to enrich the quantity of DF consumed in food. However, the poor hydroscopic capacity and high rigidity of their surface make them difficult to incorporate into food matrices. To address these issues chemical, biological, and physical treatments have been suggested. Unfortunately, chemical treatments are environmentally harmful, biological methods are detriment to the product quality, and physical treatment is complex, requiring highly specialized equipment. For these reasons a LbL methodology has been presented that sort to solve this issue. The LbL approach in question used two different layers on okara powder particles, a chitosan and then a pectin layer. This process resulted in increased water suspensibility, but the impact and industrial significance of this report is such that it requires future investigation to be realized fully.

### 4.4 Water Treatment

The use of LbL particles for water treatment has also been widely demonstrated in the sorption of organic dyes^239,240^ and heavy metal ions,^241^ and is well reviewed.^242^ A prominent example of its use in water treatment is in the stabilization of magnetic nanoparticles for water remediation, using silica and PDDA to create magnetic nanoparticle nanocomposites. This study was completed to reduce the effects of the interparticle aggregation arising from the intrinsic behaviors of magnetic nanoparticles, as well as the nanotoxicity exhibited by iron oxide magnetic nanoparticles (IOMNPs), discouraging their use ion environmental remediation. The study went on to show how the silica-PDDA-IOMNP composites were effective in achieving dye removal efficiency of ∼100%.^243^

## 5 Conclusion

The enduring fascination with LbL nanofilm deposition stems from continuous advancements in eligible materials and associated technologies, detailed in the preceding sections. These innovations signify substantial progress beyond the primitive lab-scale methodology of centrifugation. Tangential flow filtration, facilitating an automatable process, and fluidized bed techniques, achieving high yield in a scalable manner, along with the successful integration of LbL into industrial nanoparticle production demonstrated by PRINT LbL, mark significant milestones. However, despite these advancements, none of these methods comprehensively meet the requirements for a reproducible, high-yield, and automated deposition technique. Additionally, none have presented a particle coating technology versatile enough to emulate the intrinsic flexibility of LbL. This versatility must span all particle types, including nanoparticles and delicate biological matter like cells, and encompass a broad array of coating materials, while still attaining to demands for high yield, throughput, repeatability, and automation—crucial for successful industrial integration in the drug delivery and pharmaceutical sector.

Promisingly, microfluidic methods enable unparalleled control of the microenvironment and nanocoating conditions, showcasing higher reproducibility compared to larger-scale methods. However, as explored in preceding sections, microfluidics alone is only part of the solution. The integration of microfluidics with particle manipulation methods—such as magnetics, dielectrophoresis, and acoustics—proves essential. Among these, acoustic manipulation stands out as the most versatile, demonstrating suitability for all substrate types, without requirement for labeling. Despite its potential, current acoustofluidic methods in LbL coating face challenges, particularly in meeting industrial demands.

The prospect of further incorporating acoustofluidics into particle-LbL coating opens a promising pathway to address challenges encountered in previous methodologies, especially when exploring applications of acoustofluidics beyond the domain of particle coating. Illustrative instances of acoustofluidic applications, such as cell sorting and trapping, vividly demonstrate the versatility of acoustic particle manipulation across multiple dimensions and for numerous particles, encompassing both continuous and batch processes. Notably, these examples highlight the simultaneous processing of a multitude of particles, a capability that could effectively tackle the low-throughput limitations observed in existing acoustofluidic LbL technologies, provided the realization and integration of these methods into the design and fabrication of new technologies.

Another integral aspect to existing demonstrations of acoustofluidics in cell sorting and trapping is the capability for in situ monitoring and characterization, facilitating real-time assessment during LbL deposition. This feature provides researchers with unprecedented insights into the dynamics of layer formation, allowing adjustments and optimizations on-the-fly. The real-time monitoring aspect is crucial in standardizing LbL processes and protocols, enhancing reproducibility, and fostering a more thorough understanding of the intricate interactions governing the self-assembly process.

In the pursuit of sustainable practices, the impact of acoustofluidics on LbL technologies aligns with the broader initiative toward green and environmentally conscious methodologies. The efficient use of acoustic fields in manipulating particles not only enables high-throughput processing but also minimizes waste. This consideration becomes increasingly relevant as industries seek eco-friendly alternatives and regulators emphasize the importance of sustainable manufacturing practices.

As acoustofluidics continues to demonstrate versatility in manipulating a wide range of particle substrates, including delicate biological matter and nanoparticles, its impact on biomedical applications within the realm of LbL technologies becomes increasingly pronounced. The evolving landscape of biomedical applications, such as drug delivery systems and coatings for medical devices, benefits from the unique capabilities of acoustofluidic LbL coating. This technology not only holds promise in addressing specific challenges within healthcare but also aligns with regulatory considerations for the development of safe and effective medical products.

While the preceding sections have delved into the intricacies of various technologies, it is crucial to succinctly outline future perspectives and challenges to guide researchers in their upcoming endeavors. A summary of the insights presented in this review underscores key considerations for the future: 1) despite ongoing developments, none of the existing LbL methods comprehensively addresses the requirements for the seamless adoption of LbL into industrial practices; 2) within the array of available technologies, acoustofluidics stands out as an effective mechanism for versatile, label-free control of particles and cells, leveraging the microfluidic environment to provide a unique microenvironment for reproducibility and control, a combination unparalleled by alternative LbL and particle manipulation methods. These perspectives encapsulate pivotal areas for further exploration and refinement in the ever-evolving landscape of LbL technologies.

In summary, the impact of acoustofluidics on LbL technologies extends beyond its current applications in LbL coating. It showcases numerous instances where acoustofluidics excels in multiparticle manipulation, indicating its potential to revolutionize the field of LbL coating for particle matter. With demonstrated industrial readiness and theoretical capabilities in yield, homogeneity, control, and reproducibility, coupled with the integration of real-time monitoring capabilities, acoustofluidics emerges as a pivotal force shaping the future landscape of LbL technologies. This influence spans scientific, industrial, and healthcare domains, aligning with societal demands propelling scientific development. As societal needs in the drug delivery and nanomedicine sector continue to drive scientific advancements, the role of novel technologies becomes increasingly crucial in addressing these demands effectively. The most valuable tool is one that can be applied across a wide range of applications, showcasing optimal performance. In this context, acoustofluidics stands out as a ready-to-integrate, proven-yet-promising solution for LbL particle coating. Considering the criteria of effectiveness and versatility, acoustofluidics emerges as a key solution poised to contribute significantly to the evolution of LbL technologies.
